# Study of retroviral restrictions in a Tadarida bat cell line enlightens specific early blocks and TRIM5 locus multiplication

**DOI:** 10.1128/jvi.01927-25

**Published:** 2026-03-16

**Authors:** Sadayuki Ohkura, Haruka Osanai, Masumi Shimizu, Masayuki Horie, Rimpei Morita

**Affiliations:** 1 Department of Microbiology and Immunology, Graduate School of Medicine, Nippon Medical School 26367, Tokyo, Japan; 2 Department of Medicine, Nippon Medical School 26367, Tokyo, Japan; 3 Graduate School of Veterinary Science, Osaka Metropolitan University 12936 , Osaka, Japan; 4 Osaka International Research Center for Infectious Diseases, Osaka Metropolitan University 12936, Osaka, Japan; University Hospital Tübingen, Tübingen, Germany

**Keywords:** host factor, bat, TRIM5, murine leukemia virus

## Abstract

Studies on retroviral infections in bat cells have revealed lineage-dependent differences in susceptibility level, implicating the presence of retrovirus-inhibiting mechanisms in bats. However, the limited number of bat species studied has hampered the clarification of the specific underlying mechanisms. While studying the susceptibility of bat cell lines to murine leukemia virus (MLV), we identified a specific Yangochiropteran bat cell line that exhibited a primate tripartite motif-containing protein (TRIM) 5α-like MLV restriction. Here, we characterized the restrictive nature of the TRIM5α homolog in a Yangochiropteran cell line. This cell line was restrictive to gibbon ape leukemia virus (GaLV)—a phylogenetic relative to Yinpterochiropteran gammaretroviruses—and N-tropic MLV (N-MLV) dependent on the capsid (CA). A genomic search of TRIM5α-like sequences revealed four TRIM5α homologs, none of which reproduced the strong N-MLV and GaLV restriction observed in the cell line. Instead, a chimeric Yangochiropteran TRIM5α with the RING-Bbox-coiled-coil domain from a restrictive Yinpterochiropteran TRIM5α restricted B-tropic MLV independent of the proteasome. Mutational analyses of the Yangochiropteran TRIM5α revealed two responsible gain-of-function mutations in the L1 linker. One of the mutations specifically reduced the self-ubiquitination of Yangochiropteran TRIM5α, whereas its reciprocal mutation abolished N-MLV restriction by Yinpterochiropteran bat and rhesus macaque TRIM5α, highlighting the importance of this residue in the mechanism of TRIM5α-mediated restriction. Herein, we demonstrate that mutations in L1 may induce the loss of anti-MLV activity in Yangochiropteran TRIM5α, with its B30.2 domain retaining the ability to recognize the CA core and a yet-unidentified factor restricting N-MLV in the Yangochiropteran cell line.

**IMPORTANCE:**

Bats host many RNA viruses that can potentially cause spillover to humans; however, they rarely develop severe symptoms after infection, suggesting the presence of bat-specific antiviral mechanisms. Although no infectious retroviruses have been isolated from bats, we and other groups have proposed the presence of unknown retrovirus-inhibiting mechanisms in bat cells. In this study, we focused on a particular host factor (TRIM5α) in a specific Yangochiropteran bat cell line that showed resistance to gammaretroviruses; however, its TRIM5α homolog lost its antiretroviral activity, with its ligand-interacting domain retaining the ability to recognize the retrovirus capsid. We identified specific gain-of-function mutations in Yangochiropteran TRIM5α that endow the protein with gammaretrovirus-restricting activity. This finding suggests that during Yangochiropteran evolution, these mutations may have prevented Yangochiropteran TRIM5α from inhibiting gammaretroviruses. Further understanding of retrovirus-inhibiting mechanisms in bat cells would help develop new therapeutic strategies to combat retrovirus-related diseases, such as acquired immunodeficiency syndrome.

## INTRODUCTION

Bats (order Chiroptera, including two suborders: Yinpterochiroptera [formerly megabat and five microbat families] and Yangochiroptera [formerly microbats] [1, 2]) are susceptible to infection by various RNA viruses, including filoviruses, henipaviruses, rotaviruses, and beta-coronaviruses (3–8). Some bat species may also be susceptible to gammaretroviruses, as the full-length genome of Hervey pteropid gammaretrovirus (HPG) capable of producing infectious viral particles has been discovered in fecal specimens of Australian Yinpterochiropteran bats (9). However, not all bat species are equally susceptible. Our groups and other researchers have reported cell line-dependent differences in susceptibility level to murine leukemia virus (MLV), which is phylogenetically distinct from HPG (10, 11). In contrast, the studied Yinpterochiropteran bat cell lines were uniformly resistant to lentiviruses through unknown mechanisms (10, 11). These findings suggest a viral-type- and bat-lineage-dependent retroviral restriction mechanism in bat cell lines.

Studies on retroviral non-permissive cell lines have identified restrictive factors that sense viral proteins and inhibit the viral replication cycle following viral entry. Tripartite motif-containing protein 5α (TRIM5α) was first identified as a restriction factor that blocks human immunodeficiency virus type 1 (HIV-1) in primate cells and was later reported to restrict N-tropic MLV (N-MLV) (12–15). TRIM5α is a member of the TRIM protein family and has well-conserved RING (R), B-box (B), and coiled-coil (CC) domains collectively referred to as the RBCC domain. The N-terminal RING domain functions as an E3 ubiquitin ligase (16, 17), whereas the B-box2 and CC domains of TRIM5α are essential for self-oligomerization and self-association (18–25). The C-terminal portion varies depending on the family members. TRIM5α contains a B30.2 (PRYSPRY) domain, whose ligand is the retroviral capsid (CA) in the context of retroviral infection (26–30); however, its physiological ligands remain unclear. Upon recognition, TRIM5α forms a hexagonal lattice over the CA core lattice (31–34), and the L2-linker-CC domain affects CA-binding affinity/avidity by coordinating with the B30.2 domain (35). Subsequently, TRIM5α blocks viral replication before or during reverse transcription (RT) (12). *TRIM5* exists in the genomes of various mammalian species but lacks functional homologs in mice (36). The remnants of endogenous retroviruses in the genomes of various bat species suggest ancient retroviral infections (37–41), supporting the existence of retrovirus-recognizing host machinery in bats.

Another prominent MLV restriction factor in mice is Friend virus susceptibility factor 1 (Fv1) (42–47). The Fv1 alleles determine the susceptibility of mice to different tropisms of MLV. For example, Fv1^b^ restricts N-MLV, whereas Fv1^n^ restricts B-tropic MLV (B-MLV). *Fv1* is unique in that it is a part of the *gag* gene of murine endogenous retroviruses (48) and is primarily present in Muroidea rodents (49, 50). As a restriction factor, Fv1 interacts directly with the surface of MLV CA (51, 52), particularly at the amino acid residue position 110, which determines its tropism (51) and blocks viral replication during the phase between the completion of RT and integration (53). However, the origin of the most common ancestors of extant bats was estimated to be approximately 62 million years ago (Mya) (1, 54, 55), which is considerably earlier than the time of integration of an Fv1 ancestral sequence into the genome of Muroidea ancestors (>45 million years ago) (49), suggesting the absence of Fv1-related factors in bats.

While studying the susceptibility of Yangochiropteran bat cell lines to MLV infection, we found a specific *Tadarida brasiliensis* cell line (Tb1.Lu) that was significantly less permissive to MLV infection than other Yangochiropteran bat cell lines (11). In particular, Tb1.Lu cells were significantly less susceptible to N-MLV than to B-MLV, as is the case with primate cells (11), wherein TRIM5α dominantly restricts N-MLV. In the present study, we characterized *T. brasiliensis* TRIM5α (TbraTRIM5α) and demonstrated that although it was no longer responsible for N-MLV resistance in Tb1.Lu cells, TbraTRIM5α retained the ability to recognize B-MLV CA, and the two amino acids in the L1 linker affected its susceptibility to B-MLV.

## RESULTS

### MLV restriction in Tb1.Lu *Tadarida brasiliensis* cells is CA-dependent, pre-RT, dominant inhibition

In our previous study on the susceptibility of bat cell lines to retrovirus infection, we demonstrated virus type-dependent retrovirus restriction and identified Tb1.Lu cells with specific gammaretrovirus susceptibility (11). Among the Yangochiropteran cell lines investigated, only Tb1.Lu cells showed susceptibility similar to that of primate cell lines that primarily restrict N-MLV (56). To further characterize the Yangochiropteran cell line used for gammaretroviral infection, we performed the following experiments.

#### Infection of Tb1.Lu cells with gibbon ape leukemia virus

**Fig 1:**
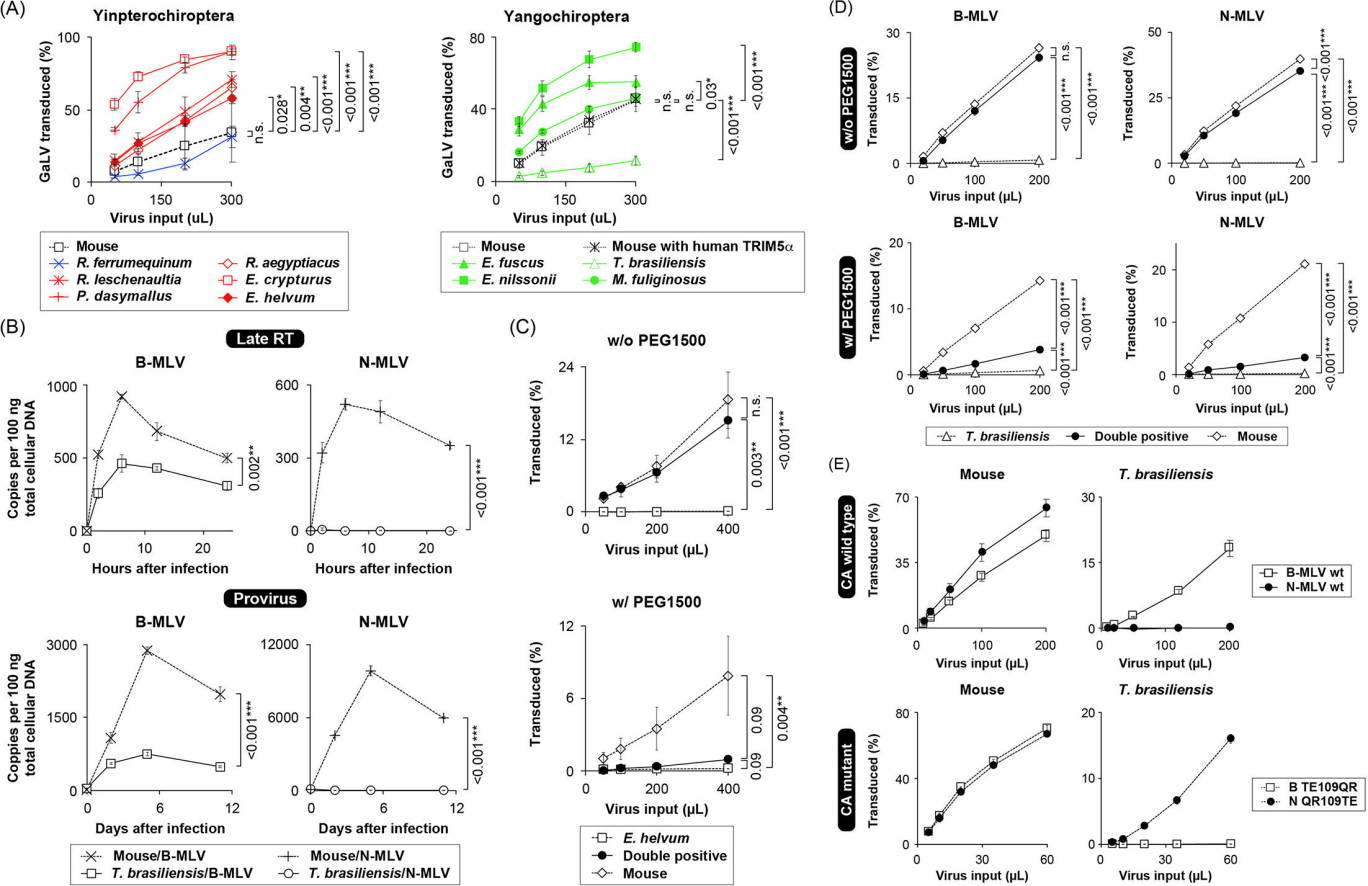
Characterization of gammaretrovirus susceptibility in a specific Yangochiropteran cell line. (**A**) Titration of GaLV in bat cell lines. Mouse MDTF (control) and bat cells were infected with increasing amounts of the GaLV tester virus expressing eGFP, and the ratio of GFP-expressing cells was analyzed using FACS. Red: Pteropodidae bat cell lines, blue: Rhinolophidae cell line, and green: Yangochiropteran cell line. (**B**) Quantification of viral genome copy numbers in MDTF or Yangochiropteran Tb1.Lu cells using qPCR. MDTF and Tb1.Lu cells were infected with MLV, and copy numbers of the late RT products and proviruses were quantified with a set of eGFP gene-specific primers and probe using total cellular DNA extracted from MLV-infected cells at 0, 2, 6, 12, and 24 h (late RT), or 0, 2, 5, and 10 days (provirus) after infection. The qPCR experiments were performed in duplicate. (**C and D**) MLV titration in PEG-treated fused cells. Permissive MDTF (eGFP +ve) and non-permissive bat (tdTomato +ve) cells were chemically fused with PEG1500 and infected with the tester MLV expressing E2Crimson. The ratio of E2Crimson-expressing cells was measured using FACS. MDTF cells were fused with Yinpterochiropteran ZFBK13-76E (**C**) or Yangochiropteran Tb1.Lu (**D**) cells. (**E**) The exchange of amino acid residues at positions 109/110 between B- and N-MLV CA reversed the MLV susceptibility of Tb1.Lu cells. Each experiment was independently repeated at least three times. The obtained data at each sampling in the biological replicates were averaged, and data variability is represented by an error bar (mean ± SD). Statistical significance was assessed using ANOVA (*P* < 0.05 was considered significant). Dunnett’s post hoc comparisons between the control (“Empty”) and the subject groups were performed for the data obtained from the cells with the highest viral inoculum. Results with *P* values of * <0.05; ** <0.01; *** <0.001 were considered statistically significant.

A group of HPG-related gammaretroviruses, including gibbon ape leukemia virus (GaLV) and koala retrovirus, is phylogenetically distinct from MLV (9). In this context, we evaluated the permissiveness of Tb1.Lu cells to a biologically relevant GaLV. Compared to MLV, which was restricted in Yinpterochiropteran cells due to the expression of TRIM5α homologs (10, 11), all of the bat cell lines studied, except Tb1.Lu cells, were susceptible to GaLV infection (Fig. 1A). This finding suggests that Tb1.Lu cells were less permissive to both MLV- and HPG-related gammaretroviruses than the other Yangochiropteran cells.

#### Measuring MLV RT products in *T. brasiliensis* cells

To identify the stage at which MLV infection was blocked in Tb1.Lu cells, we examined the MLV RT products. We quantified the expression of the eGFP gene as a late RT product using quantitative polymerase chain reaction (q-PCR) to avoid the background detection of endogenous bat retroviruses (11). Specific detection of MLV late RT products was verified using *Mus dunni* tail fibroblast (MDTF) cells, which are Fv1-null and lack functional homologs of primate TRIM5α (36, 57) (Fig. 1B). In B-MLV-infected Tb1.Lu cells, eGFP gene expression was detected but at significantly low levels, whereas it was almost absent in N-MLV-infected Tb1.Lu cells (Fig. 1B), suggesting that N-MLV failed to complete RT. We also examined viral genome integration in multi-passaged MLV-infected Tb1.Lu cells. Compared with that in successfully integrated cells, in which the viral genome must be retained as a provirus, viral genome integration was lost in virus-inhibiting cells via degradation and/or dilution after multiple passages when the virus replication cycle was blocked before integration into the host genome (11). eGFP gene expression was barely detected in N-MLV-infected, multi-passaged Tb1.Lu cells (Fig. 1B), indicating N-MLV restriction before integration. In B-MLV-infected, multi-passaged Tb1.Lu cells, eGFP gene expression was significantly reduced compared with that in infected MDTF cells (Fig. 1B), consistent with the reduction in late RT product levels. Therefore, the MLV infection was interrupted before proviral integration into Tb1.Lu cells.

#### Infection of heterokaryonic cells with MLV

To investigate the presence of MLV-inhibiting factors in bat cells, we infected the heterokaryons of permissive (eGFP-expressing MDTF cells) and non-permissive (tdTomato-expressing bat cells) cells with MLV. Inhibition of viral infection in heterokaryons implies the presence of a virus-inhibiting host factor (11). Cells were fused using polyethylene glycol (PEG), followed by infection with the E2Crimson-expressing tester MLV. The E2Crimson-positive cell proportion in the double-positive (DP) cell fraction represents MLV infectivity in heterokaryons. In the control, heterokaryons of Yinpterochiropteran *Eidolon helvum* cells, which express an MLV-restricting TRIM5α homolog (11), and MDTF cells showed significantly reduced susceptibility to N-MLV (Fig. 1C), in contrast to their high susceptibility to Moloney MLV (11), validating the experimental system. The fusion of MDTF with Tb1.Lu cells conferred resistance to both B- and N-MLV (Fig. 1D), which was consistent with a significant reduction in the synthesis of RT products in Tb1.Lu cells (Fig. 1B). The MLV susceptibility of the heterokaryons was not as low as that of the parental bat cells, likely because of the small fraction of unfused doublets in the DP population. DP cells in the PEG-untreated controls showed doublets, which were not excluded from fluorescence-activated cell sorting (FACS) analysis because of their large sizes, similar to those of the PEG-treated heterokaryocytes. These findings suggest the presence of MLV-inhibiting factors in bat cells.

#### Introduction of point mutations in the MLV CA

To investigate the target of the antiviral activity in Tb1.Lu cells, we introduced mutations in CA that exchanged the B- and N-tropisms of MLV. Specifically, threonine (T) and glutamate (E) at positions 109 and 110 were substituted with glutamine (Q) and arginine (R), respectively, in the B-MLV background (B-MLV TE109QR), and vice versa (N-MLV QR109TE) (51). In Tb1.Lu cells, these CA mutations reversed cell susceptibility; N-MLV was no longer restricted, and B-MLV showed reduced infectivity (Fig. 1E), suggesting CA-dependent mechanisms. Collectively, these results are reminiscent of TRIM5α-mediated MLV restriction in primate cells.

### TbraTRIM5α is partially responsible for MLV restriction

To investigate the involvement of TRIM5α in MLV restriction in Tb1.Lu cells, we cloned the cDNA of Yangochiropteran TRIM5α homologs using primers designed to amplify TRIM5α transcripts across the Yangochiroptera genus based on a public database (NCBI Nucleotide) (11). Using these primers, we cloned three *T. brasiliensis* TRIM5α (TbraTRIM5α) cDNAs (11). Sequencing analysis of the newly cloned cDNAs revealed that only variant 2 (v2) encoded full-length TRIM5α. Hereinafter, v2 represents TbraTRIM5α (GenBank #OP272523.1). As v1 encodes only the N-terminal RBCC domain, it was used as a control that did not recognize the retroviral CA core. v3 was excluded from this study owing to the lack of almost all of the functional motifs (Fig. S1A).

**Fig 2:**
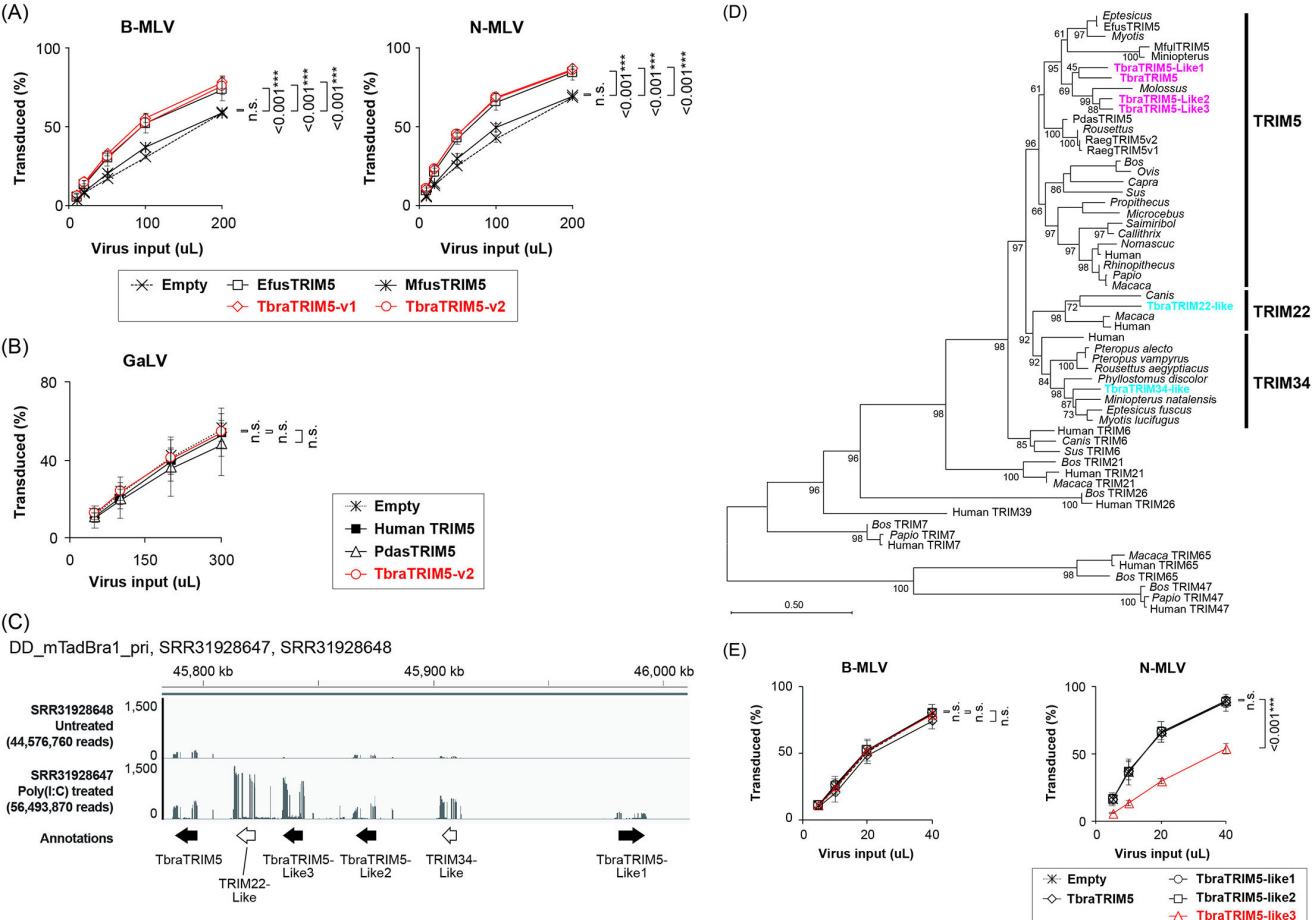
Yangochiropteran TRIM5α does not restrict gammaretroviruses. TRIM5α cDNAs cloned in Yangochiropteran EfK3B, Tb1.Lu, and YubKT-1 cell lines were stably transduced in MDTF, and MLV restriction in the resultant transduced cells was examined. (**A, B, E**) Titration of MLV (**A and E**) and GaLV (**B**) in Yangochiropteran TRIM5α-expressing MDTF. The GFP-positive cell ratio was measured using FACS 48 h after infection. Each titration experiment was repeated three times independently. For statistics, see the legend of Fig. 1. (**C**) Mapped read coverages of TRIM5α-related genes obtained from publicly available RNA-Seq data of untreated or poly(I:C)-treated Tb1.Lu cells (BioProject: PRJNA1207558; SRR31928647, and SRR31928648) (Table S1). (**D**) Phylogenetic relationships between mammalian TRIM5α, including bat TRIM5α and other TRIMs related to mammalian TRIM5α. The newly identified TRIM5α genes are highlighted in magenta. A maximum-likelihood (ML) phylogenetic tree was constructed based on the full-length amino acid sequences of TRIMs. Note that additional transcripts in the TRIM5α loci whose expression levels were increased by poly(I:C) treatment are TRIM22-like and TRIM34-like (**C**). They are also included in panel **D** (highlighted in cyan).

Yangochiropteran TRIM5α cloned from a representative cell line from each Yangochiroptera family (*Eptesicus fuscus* from Vespertillonidae, *T. brasiliensis* from Molossidae, and *Miniopterus fuliginosus* from Miniopteridae) (11) was stably transduced into MDTF cells (Fig. S2A). In contrast to that of Yinpterochiropteran TRIM5α, which restricts HIV-1 and MLV (11), the expression of the Yangochiropteran TRIM5αs had minimal effects on MLV or GaLV infectivity (Fig. 2A and B).

A search for TbraTRIM5α-like sequences in the *T. brasiliensis* genome (NCBI Sequence Read Archive [SRA], DD_mTadBra1_pri) yielded three putative TbraTRIM5α-like sequences that were located close proximal to the *TbraTRIM5a* gene (Like1, Like2, and Like3 paralogs; Fig. 2C). Each sequence fully encoded the *TRIM5a* gene (Fig. 2D and S1B). To amplify cDNAs, the isoform-specific primers were designed based on the obtained sequences. While preparing this manuscript, Owolabi IJ et al. registered the RNA-Seq data of Tb1.Lu cells, untreated or treated with poly(I:C), in the NCBI SRA database (BioProject: PRJNA1207558) (58). A comparison of the expression levels of TRIM5α transcripts (SRR31928647 and SRR31928648) revealed that while the TbraTRIM5α and Like2 transcripts were constitutively expressed in untreated Tb1.Lu cells, their expression levels were increased by poly(I:C) treatment. In contrast, the expression of Like1 and Like3 transcripts was remarkably more increased than TbraTRIM5α and Like2 by poly(I:C) treatment in Tb1.Lu cells (Fig. 2C). Accordingly, we attempted to amplify the cDNAs of TbraTRIM5α-like transcripts from untreated and poly(I:C)-treated Tb1.Lu cells, and the cDNAs of all paralogs were successfully cloned (Fig. S1B and S3D). Although quantitative RT-PCR analysis could not be performed owing to the high nucleotide sequence similarities between the Like2 and Like3 transcripts, we noticed that poly(I:C) treatment drastically augmented Like1 mRNA expression in Tb1.Lu cells (Fig. S3B).

The TbraTRIM5α-Like1, -Like2, and -Like3 cDNAs were stably transduced in MDTF cells, and MLV infectivity was assayed in stable MDTF cells (Fig. 2D). Only Like3 significantly reduced N-MLV infectivity in MDTF cells (Fig. 2E), although the reduction of the infectivity was approximately twofold and did not reproduce the strong reduction in N-MLV infectivity in Tb1.Lu cells (>30-fold) (11). Therefore, Like3 could not be a dominant factor of the N-MLV restriction. In stable MDTF cells, all Yangochiropteran TRIM5α proteins were diffused in the cytoplasm, similar to Yinpterochiropteran and primate TRIM5α (Fig. S2B), ruling out the possibility of their aberrant intracellular localization causing poor MLV inhibition. Collectively, TRIM5α proteins do not appear to be fully responsible for N-MLV restriction in Tb1.Lu cells.

### L1-Bbox2 region determines MLV restriction specificity

**Fig 3:**
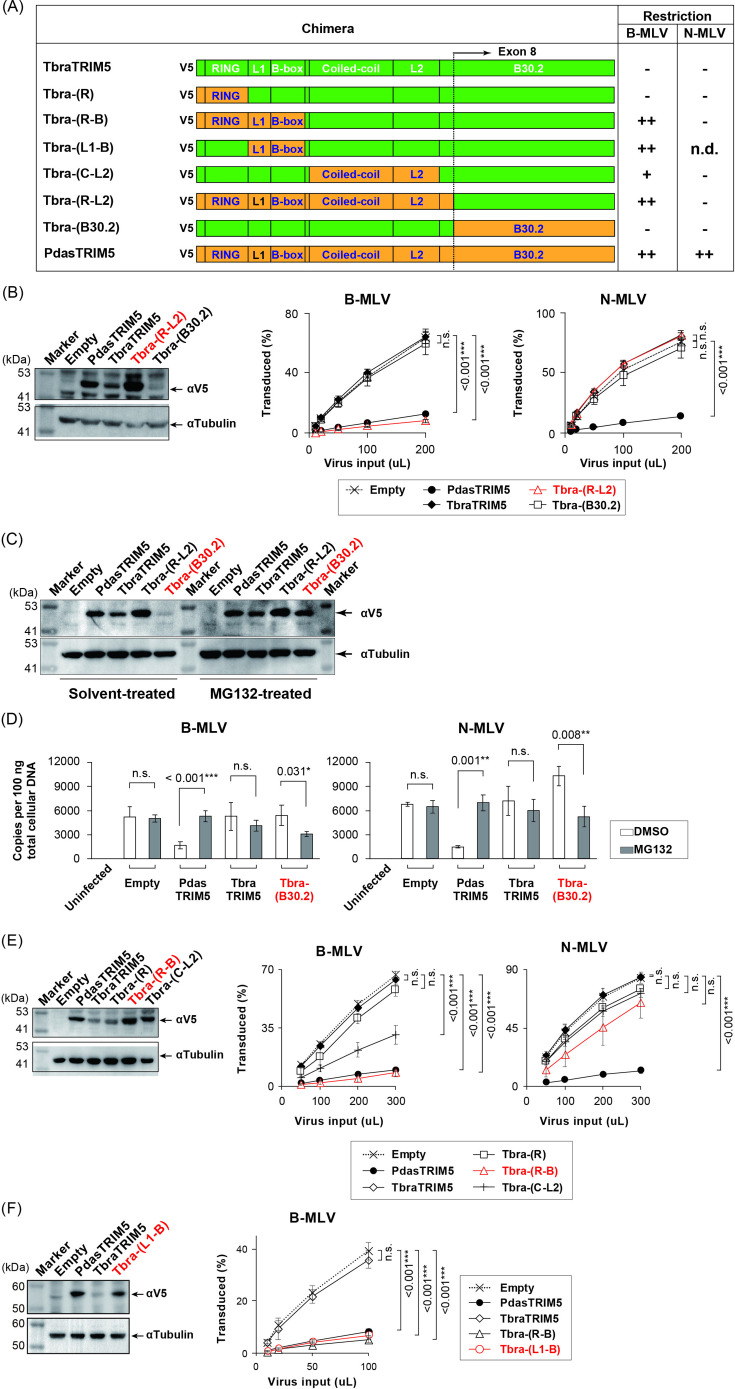
MLV-restricting activity of bat TRIM5α chimeras. (**A**) Schematic structures of bat TRIM5α chimeras constructed in this study (left column) and their restriction (right columns). The characters within parentheses following “Tbra” indicate the PdasTRIM5α-derived fragment inserted in the TbraTRIM5α background. MLV restriction is presented as a fold reduction of infectivity in MDTF cells expressing each TRIM5α construct relative to that in MDTF cells expressing the empty vector, based on the results shown in panels B, E, and F. −, <2; +, 2–5; ++, >5. (**B, E, F**) Protein expression analysis using western blotting and titration of MLV in stable MDTF cells expressing bat TRIM5α and their chimera. Shown are the exchanges of the B30.2 domain only (**B**) and the insertion of each RBCC motif of PdasTRIM5α in the TbraTRIM5α background (**E and F**). For the titration experiments and the statistics, see the legend of Fig. 1. (**C**) Western blotting to detect TRIM5α in the cell lysate from TRIM5α-expressing MDTF cells treated with either the proteasome inhibitor MG132 or a solvent (DMSO) using a V5-tag-specific mAb. (**D**) qPCR to measure the copy numbers of the MLV late RT products in bat TRIM5α-expressing MDTF cells with or without MG132 treatment as described in the legend of Fig. 1B. Data represent mean ± SD of the results obtained from three independent experiments. Statistical significance was assessed between the MG132-untreated (open) and treated (gray) groups using the Student’s *t*-test. Results with *P* values of * <0.05; ** <0.01, and *** <0.001 were considered statistically significant.

Considering the primate TRIM5α-like, N-MLV-preferred restriction pattern in Tb1.Lu cells, we sought to determine the reason for the inability of TRIM5α to restrict MLV in Tb1.Lu cells using the first identified TRIM5α variant (TbraTRIM5α) for further characterization. We expected that the CA-interacting B30.2 domain might render TbraTRIM5α inactive under MLV restriction because non-restrictive TRIM5α can become restrictive by replacing its B30.2 domain with that of its restrictive counterpart (26, 59, 60). The B30.2 domain (exon 8) was exchanged between *Pteropus dasymallus* (Pdas)TRIM5α and TbraTRIM5α to create chimeric TRIM5α: Tbra-(R-L2) and the reciprocal Tbra-(B30.2) (Fig. 3A). In stable MDTF, Tbra-(R-L2) selectively restricted B-MLV, whereas the reciprocal Tbra-(B30.2) had a poor effect on the infectivity of both MLV types (Fig. 3B). This finding suggests that the RBCC domain, and not the B30.2 domain, is responsible for determining MLV susceptibility in bat TRIM5α.

The lower expression of TbraTRIM5α and Tbra-(B30.2) than that of the other TRIM5α proteins (Fig. 3B, left) may reflect their proteasomal degradation. Upon inhibition of the proteasome with its specific inhibitor, MG132, the protein levels of non-restricting TbraTRIM5α and Tbra-(B30.2) increased to levels comparable to those of restrictive PdasTRIM5α and Tbra-(R-L2) (Fig. 3C), indicating that TRIM5α with the RBCC domain from non-restricting TbraTRIM5α was degraded by the proteasome. To investigate the effect of proteasome inhibition on MLV restriction, the copy numbers of viral RT products were compared between untreated and MG132-treated MDTF cells (Fig. 3D). Although the levels of the RT products of MLV in MDTF cells expressing non-restricted TbraTRIM5α were not significantly affected by MG132 treatment, the expression of MLV RT products was rescued by proteasomal inhibition in MDTF cells expressing restricted PdasTRIM5α, suggesting no association between TbraTRIM5α protein accumulation and MLV restriction. In contrast, the levels of RT products of both MLVs were significantly reduced by MG132 treatment in MDTF cells expressing Tbra-(B30.2). These observations suggest that, in contrast to the parental TbraTRIM5α, protein accumulation is associated with MLV restriction by Tbra-(R-L2) and Tbra-(B30.2) chimeras.

To further characterize the unexpected restriction of B-MLV by Tbra-(R-L2) (Fig. 3B), we replaced each functional motif within the RBCC domain with the corresponding motif from PdasTRIM5α in the background of TbraTRIM5α either individually or in combination: Tbra-(R), Tbra-(R-B), Tbra-(C-L2), and Tbra-(L1-B) (Fig. 3A). Although the protein level of Tbra-(R) was as low as that of TbraTRIM5α, the levels of Tbra-(R-B) and Tbra-(L1-B) proteins were comparable to the level of PdasTRIM5α (Fig. 3E and F), suggesting that the L1-B region of PdasTRIM5α rescued the protein level of TbraTRIM5α. Replacement of the RING-Bbox2 or L1-Bbox2 regions in Tbra-(R-B) or Tbra-(L1-B), respectively, effectively blocked B-MLV infection to an extent similar to that by PdasTRIM5α (Fig. 3E and F). Another chimera, Tbra-(C-L2), significantly reduced B-MLV infectivity; however, this reduction was not as effective as that by PdasTRIM5α (Fig. 3E). These results suggest that L1-Bbox2 plays a role in B-MLV restriction. N-MLV restriction was not affected by the expression of any of the chimeras (Fig. 3A). Therefore, we focused on B-MLV restriction in this study.

### Serine residues in L1 determine restriction activity

**Fig 4:**
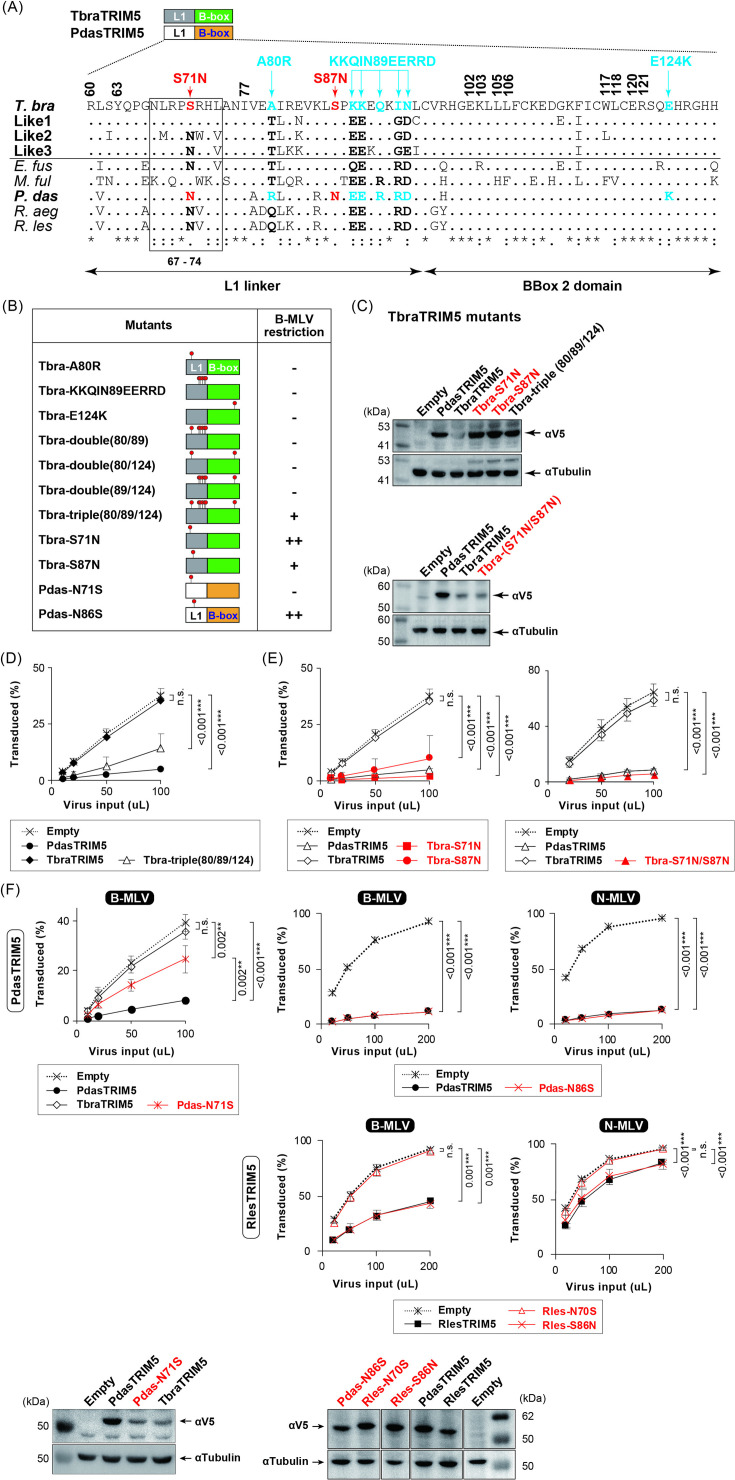
Effects of point mutations introduced in L1-Bbox2 of TbraTRIM5α and Yinpterochiropteran TRIM5α on MLV restriction. (**A**) Amino acid alignment of L1-Bbox2 of bat TRIM5α was studied. Each dot indicates the identical amino acid to TbraTRIM5α. The positions of the amino acid residues reportedly involved in rhTRIM5α-mediated HIV-1 restriction (20, 22, 62) are indicated at the top of the alignment, highlighted with black bold figures. A stretch of six amino acids involved in the RING-RING interaction and higher-order association (62) is boxed (positions 67–74 in TbraTRIM5α). The residues mutated in this study to modify the charges and the exchange of S-to-N are highlighted in cyan and red, respectively, with vertical arrows. (**B**) Schematic structures of bat TRIM5α mutants constructed in this study (left column) and their restriction (right columns). Approximate positions are highlighted with red pins. B-MLV restriction is presented as a fold reduction as described in the legend of Fig. 3A, based on the results shown in panels **D**, **E**, **F**, and Fig. S4A. (**C**) Protein expression analysis of bat TRIM5α mutants using western blotting. TRIM5α proteins on the membrane were probed with a V5-tag-specific mAb. (D, E, and F) Titration of MLV in MDTF cells stably expressing TbraTRIM5α with either triple charge-changing point mutations (**D**) or S-to-N mutations (**E**) and Yinpterochiropteran TRIM5α with S/N exchanging mutations (**F**) as described in the legend of Fig. 1. Panel **F** also includes western blotting results of PdasTRIM5α and RlesTRIM5α mutants using a V5-tag-specific mAb. Their original western blot images are included in Fig. S4C. The titration (**D, E, and F**) experiments were repeated three times independently. For statistics, see the legend of Fig. 1.

To specify the sites responsible for B-MLV restriction in L1-Bbox2, the protein sequences of the region were compared between bat TRIM5α (Fig. 4A). Mutational analyses of primate TRIM5α have identified residues involved in antiretroviral activity (20, 61, 62), and they are identical in Tbra and PdasTRIM5α (Fig. 4A; residues with black bold figures). We first modified the charges at positions 80, 89–95, and 124 in TbraTRIM5α (Fig. 4A; cyan and Fig. 4B) based on a recent report that charge reversal in L1-Bbox2 affected the viral-restriction ability of primate TRIM5α (22). Among the resultant mutants, only the triple mutant protein significantly restricted B-MLV with accumulation levels similar to those of PdasTRIM5α (Fig. 4C and D; Fig. S4A), although the reduction in infectivity was weaker than that observed with PdasTRIM5α (Fig. 4D). Therefore, charge modifications in L1-Bbox2 did not fully convert TbraTRIM5α into a restrictive form.

Next, we replaced serine (S) residues at positions 71 and 87 with asparagine (N) in TbraTRIM5α (S71N and S87N, respectively) (Fig. 4A; red). S71 is part of a six-amino acid stretch (boxed in Fig. 4A) reportedly involved in the RING-RING interaction and self-ubiquitination of rhesus macaque (rh)TRIM5α (62). These two mutations enabled TbraTRIM5α to restrict B-MLV to a level comparable to that of PdasTRIM5α (Fig. 4E) and drastically rescued TbraTRIM5α protein level in transduced cells separately (Fig. 4C, upper panel), but not in combination (Fig. 4C, lower panel). Consistently, the reciprocal Yinpterochiropteran TRIM5α mutants (Pdas-N71S and Rles-N70S) lost their ability to inhibit both MLV types, with a significant reduction in the level of PdasTRIM5α (Fig. 4F), but not that of RlesTRIM5α (Fig. S4C), supporting the observation that Yinpterochiropteran TRIM5α restricted both MLV types (Fig. 3B) (11). The mutation at position 86 did not affect MLV restriction by Yinpterochiropteran TRIM5α (Fig. 4F). As expected, neither mutation affected the anti-MLV activity nor the protein levels of non-restricting RaegTRIM5α (Fig. S4C). In contrast, TbraTRIM5α’s ability to restrict N-MLV was unaffected by the S71N mutation (Fig. S4B and E), suggesting a B-MLV-specific effect on infectivity by the S71N mutation.

To assess whether the effects of S-to-N mutations on MLV restriction were functionally conserved among Yangochiropteran TRIM5α proteins, the S or N residues at positions 71 and 87 were replaced with N or S, respectively, in Yangochiropteran TRIM5α. None of the resultant mutants were restrictive to B-MLV (Fig. S4D), suggesting that the acquisition of B-MLV-restricting activity through S-to-N mutations was specific to TbraTRIM5α.

### Residue N71 is important for rhTRIM5α-mediated N-MLV restriction

**Fig 5:**
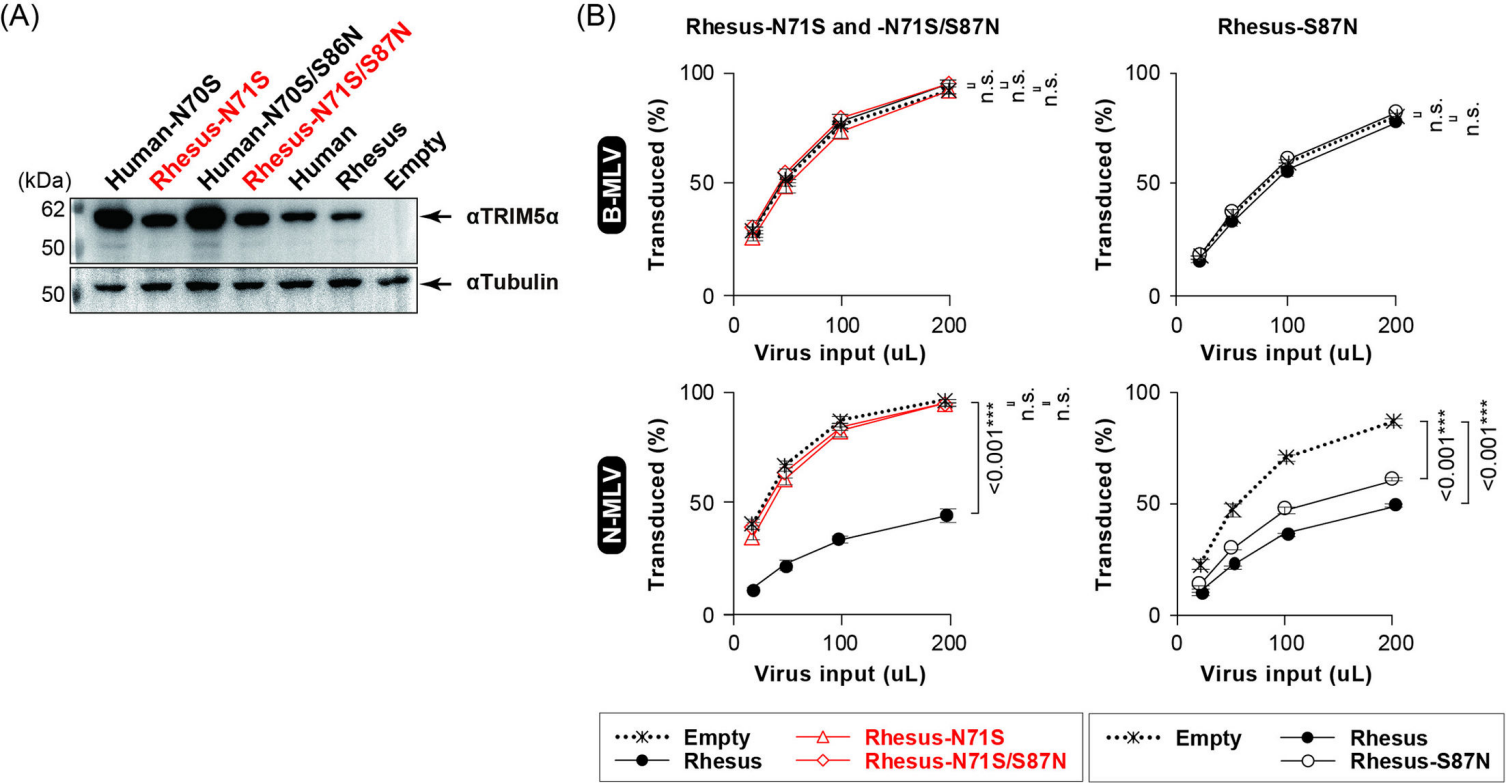
Effect of the N71S mutation on primate TRIM5α-mediated restriction. (**A**) Protein expression of primate TRIM5α and its mutants in stable MDTF cells as detected using western blotting. The TRIM5α proteins on the membrane were probed with a TRIM5α-specific mAb. (**B**) Effects of the S-to-N mutation at positions 71 and 87 on the rhTRIM5α-mediated restriction. MDTF cells stably expressing rhTRIM5α mutants were titrated with B- and N-MLV as described in the legend of Fig. 1. For statistics, see the legend of Fig. 1.

Residue N71 was also conserved in primate TRIM5α (Fig. S5A). To investigate whether modifying N71 and S87 [N70 and S86 in human (hu)TRIM5α] affected the ability of primate TRIM5α to restrict N-MLV, they were mutated to S and N, respectively, in hu and rhTRIM5α (Fig. 5A). N-MLV restriction was alleviated by the N71S mutation, but not by the S87N mutation, in rhTRIM5α (Fig. 5B and S5B). Unlike TbraTRIM5α, primate TRIM5α with either mutation did not inhibit B-MLV (Fig. 5B and S5B). These observations suggest that the importance of N71 in MLV restriction is not limited to TbraTRIM5α and that the effect of N71 on MLV restriction depends on other residues in TRIM5α.

We further investigated the effect of the N71S mutation on lentiviral restriction in human U-2 OS cells stably expressing primate TRIM5α (Fig. S5C). Titration of HIV-1 and SIVmac in stable cells showed that the N71S mutation in RhTRIM5α slightly increased the SIVmac infectivity (Fig. S5D). Although this increase in infectivity was statistically significant, the extent of increase was less than twofold; therefore, we concluded that the effect of N71 on rhTRIM5α-mediated restriction is specific to N-MLV.

## DISCUSSION

In this study, we characterized a full-length variant of TRIM5α homologs predominantly expressed in a specific Yangochiropteran *T. brasiliensis* cell line, which has exceptional MLV-restriction ability and an obvious N-MLV preference. However, none of the TRIM5α homologs from this cell line fully reproduced the strong N-MLV restriction in Tb1.Lu cells. This observation differentiates TbraTRIM5 from Yinpterochiropteran and primate TRIM5α, whose MLV restriction phenotypes reflect those of the cell lines from which they originated. Therefore, although the CA-dependent and proteasome-independent MLV resistance of *T. brasiliensis* cells (Fig. 1E and 3D) was not attributed to TRIM5α, we investigated the restrictive potential of TbraTRIM5α.

TbraTRIM5α may still possess the ability to recognize the B-MLV CA core, as the chimeric constructs containing the CA-binding B30.2 domain from TbraTRIM5α restricted B-MLV [Tbra-(R-L2), Tbra-(R-B), and Tbra-(L1-B)] (Fig. 3A). However, the direct binding of TbraTRIM5α to the CA core requires further investigation. While TbraTRIM5α was the only TRIM5α among the Yangochiropteran TRIM5α homologs that acquired B-MLV restriction through single mutations in L1, especially the S71N mutation (Fig. 4E; Fig. S4D), the reciprocal mutation abolished the N-MLV restriction mediated by Yinpterochiropteran and simian TRIM5α (Fig. 4F and 5B; Fig. S4E ). These observations suggest that the importance of N71 in MLV restriction is not limited to Yangochiropteran TRIM5α. This residue may participate in TRIM5α-mediated MLV restriction in concert with other unidentified residues, as the N/S mutation did not reduce the protein level of RlesTRIM5α_N70S (Fig. S4C), nor did it eliminate N-MLV restriction by huTRIM5α_N70S (Fig. S5B). Taken together, this study revealed two novel residues in L1 that affect the MLV restriction activity of TRIM5α.

A genomic search for TRIM5α-like sequences in the *T. brasiliensis* genome revealed four TRIM5α-coding genes, forming the TRIM5α locus (Fig. 2C). This observation suggests that the TRIM5α gene was duplicated several times in the *T. brasiliensis* genome, in line with a previous publication reporting several other duplication events of TRIM5 during Chiroptera evolution (63). The mRNA expression of Like1 was significantly upregulated by poly(I:C) treatment (Fig. S3D), implying its potential mRNA-induction ability in response to RNA virus infection. However, the viral targets of TRIM5α-mediated restriction in Yangochiropteran bats remain unclear. Considering the high susceptibility of Yinpterochiropteran cell lines to HPG-related GaLV (Fig. 1A), the potential circulation of HPG-like viruses among Yinpterochiropteran bats may have driven the evolution of mechanisms that inhibit genetically related MLV-like viruses in the *Pteropus/Rousettus* lineage.

Considering the earlier origin of the common ancestors of bats than the integration of an Fv1 ancestral sequence into the Muroidea genome, the presence of Fv1 is unlikely in bats (see Supplemental Results). Nevertheless, it is possible that a protein encoded by the open reading frame of other types of endogenous retroviruses can compensate for the absence of Fv1-related factors in bats. Extensive genomic exploration of the *Tadarida*-specific endogenous retroviruses involved in MLV restriction may explain the specific resistance of *T. brasiliensis* cells to gammaretroviruses.

Although a comparative analysis of Yinptero and Yangochiropteran TRIM5α revealed two residues in L1 of TRIM5α that are responsible for MLV restriction, certain limitations were noted. First, the limited availability of Yangochiropteran cell lines might limit the identification of MLV-restricting TRIM5α gene copies. It remains unclear whether the observed low susceptibility of *T. brasiliensis* cells to gammaretroviruses is a shared characteristic among *Tadarida* spp. or is attributed to its organ-specific features (11), hampering the generalizability of our findings. Second, the residues that participate in MLV restriction in concert with N71 and N87 in TbraTRIM5α are still unknown. Third, the TbraTRIM5α paralog proteins were faintly stained using immunocytostaining even in stably transduced cells (Fig. S2B), possibly reflecting rapid proteasomal degradation, which may have caused poor antiviral activities of TbraTRIM5-Like1 and -Like2 (Fig. 2E). Lastly, the acquisition mechanisms of B-MLV-restricting ability by TbraTRIM5α upon single-amino acid modifications in L1 remain unclear. For example, S/N exchange at positions 71 and 86 rescued TbraTRIM5α protein accumulation individually, but not in combination (Fig. 4C). However, protein accumulation was insufficient for TbraTRIM5α to restrict B-MLV, as observed for double-charge-modifying mutants (Fig. S4A). Moreover, examination of self-ubiquitination of TbraTRIM5α and its derivatives revealed that Pdas and TbraTRIM5α were similarly ubiquitinated, and the S71N and S86N mutations differentially influenced self-ubiquitination of TbraTRIM5α (see Supplemental Results; Fig. S6). Therefore, neither self-association nor self-ubiquitination activity correlated with MLV restriction. In this context, although there has been no evidence backing the claim that an adaptor-like TRIM5α-binding protein is required for the antiviral function of TRIM5α, we cannot exclude the possibility that a Tadarida-specific factor could affect the specificity of TbraTRIM5α-mediated MLV restriction.

In summary, our study highlights the specific gammaretrovirus-restriction abilities of the Yangochiropteran cell line and TRIM5α. MLV restriction may involve multiple determinants of the TRIM5α sequence, making it challenging to clearly describe the antiviral mechanisms of bat TRIM5α. However, our study revealed the distinctive features of bat TRIM5α in the context of MLV restriction. Further investigations into retroviral restriction in bat cells will elucidate the precise function of bat TRIM5α and reveal yet-undiscovered retroviral restriction mechanisms.

## MATERIALS AND METHODS

### Cells

Human HeLa and African green monkey Vero cells were maintained in modified Eagle’s medium (MEM) (Nacalai Tesque) supplemented with 10% fetal bovine serum and 1× penicillin/streptomycin (Nacalai Tesque). Mouse MDTF cells were maintained in Dulbecco’s MEM (Nacalai Tesque) supplemented with 10% fetal bovine serum and 1× penicillin/streptomycin. Bat cell lines and human embryonic kidney (HEK) 293FT cells (Thermo Fisher Scientific) were maintained as previously described (Table 1 [11]). The cells transduced with the gene of interest were selected using 1 mg/mL geneticin (Nacalai Tesque) for 10 days.

**TABLE 1:** Bat cell lines used in this study

Cell line	Species
Yinpterochiroptera	
FBKT-1	*Pteropus dasymallus*
DemKT-1	*Rousettus leschenaultia*
R06E	*Rousettus aegyptiacus*
ZFBK11-97	*Epomophorus crypturus*
ZFBK13-76E	*Eidolon helvum*
BKT-1	*Rhinolophus ferrumequinum*
Yangochiroptera	
YubKT-1	*Miniopterus fuliginosus*
Tb1.Lu	*Tadarida brasiliensis*
EfK3B	*Eptesicus fuscus*
EnK	*Eptesicus nilssonii*

### Plasmid DNA

Bat TRIM5α was cloned from bat cells into the retroviral gene delivery vector pLGatewaySN as previously described (11). To construct chimeric TRIM5α, PCR-amplified gene fragments were assembled using the Gibson Assembly protocol, as described previously (11). Point mutations were introduced using the inverse PCR method, as described previously (11), except that Q5 Hot-start High-fidelity DNA polymerase (New England Biolabs) was used. The oligonucleotide primers used in this study are listed in Table 2. The nucleotide sequences of the insert-vector boundaries and point mutations were verified using Sanger sequencing in both directions.

**TABLE 2:** Oligonucleotide sequences of the PCR primers used in this study

Primer		Direction	Sequence (5′–3′)
Cloning of TbraTRIM5-like transcripts in Tb1.Lu			
Yangochiropteran TRIM5		F	ATGGCTTCRGGAATYSTG + vector-specific sequence
		R	TTAAGARCYKGGAGAACACAG + vector-specific sequence
TbraTRIM5-Like1		F	ATGGCTTCGGGAGTCCTG + vector-specific sequence
		R	TCACTTTGTTGGATGCACC + vector-specific sequence
TbraTRIM5-Like2/3		F	ATGGCTTCAGGAGTCCTG + vector-specific sequence
		R	TTAAGAGCTTGGGGAACATAG + vector-specific sequence
Introduction of point mutations and addition of Flag-tag			
Efk3B-N71S		F	CTGCGTCCCAGTCGACATGTG
		R	GTTCTCAGGCTGGTAACTG
Efk3B-S87N		F	GTGAAGTTGAACCCACAGGAG
		R	CTCCCTAAGCGTCTCCAC
YubKT-S71N		F	CTGCAGCCTAATTGGAAATTG
		R	TTTCTCAGGCTGGTAATTG
YubKT-S87N		F	GTCAAGCTGAACACAGAGGAG
		R	TCTTTGAAGTGTCTCGACTATG
FBKT-N71S		F	CTACGGCCTAGTCGACATCTG
		R	GTTTCCAGGTTGGTAACTG
Tb1Lu-S71N		F	CTGCGGCCTAATCGGCATTTG
		R	GTTCCCAGGCTGGTAACTTAG
Tb1Lu-S87N		F	GTCAAGTTGAACCCAAAGAAG
		R	CTCCCTAATCGCCTCTAC
Tb1Lu-Bbox A80R		F	CATAGTAGAGAGGATTAGGGAGGTCAAGTTG
		R	TTGGCCAAATGCCGACTA
Tb1Lu-Bbox KK89EE		F	GAAGAGAGATCTCTGTGTGCGCCATGGA
		R	CGCTCCTCCTCTGGGCTCAACTTGACCTC
Tb1Lu-Bbox E124K		F	GCGGTCTCAAAAGCACCGTGG
		R	TCACAGAGCCAGCAAATGAACTTC
Human-S86N		F	GTCAAGTTGAACCCAGAGGGG
Rhesus-S87N		F	GTCAAGTTGAACCCAGAAGAG
Human/rhesus TRIM5 S86/87N		R	CTCCCTGAGCTTCTCCAC
N-FLAG		F	GATGATGATAAAGGAGGTGGAGGTAGTATG
		R	ATCTTTATAATCCATGATAACGAATTCCGG
Chimera construction			
Tb1Lu-(R-L2)	Insert	F	ATGTGCGGCGCTACTGGGTTCACGTGACCCTGGATCCTC
		R	AGCCGGATCCTCGAGTTATCTTAAGAGCTGGGAGAACACAG
	Vector	F	GATAACTCGAGGATCCGGCTG
		R	AACCCAGTAGCGCCGCAC
Tb1Lu-(B30.2)	Insert	F	ATGCGCAACGCTACTGGGTTCACGTGATCCTGGATATTC
		R	AGCCGGATCCTCGAGTTATCTTAAGAGTTTGGAGAAGAC
	Vector	F	GATAACTCGAGGATCCGGC
		R	AACCCAGTAGCGTTGCGC
Tb1Lu-(R)	Insert	F	AGGCGCCGGAATTCGTTATCATGGGCAAACCGATTCCGAACC
		R	CCAGGCTGGTAACTTAGCCTGCACACCGGGCAGCTGCT
	Vector	F	AGGCTAAGTTACCAGCCTG
		R	GATAACGAATTCCGGCGC
Tb1Lu-(R-B)	Insert	F	AGGCGCCGGAATTCGTTATCATGGGCAAACCGATTCCGAACC
		R	ACCTCCTCCATGAGGAATGTGTGGTGACCACGATGCTTC
	Vector	F	ACATTCCTCATGGAGGAG
		R	GATAACGAATTCCGGCGC
Tb1Lu-(L1-B)	Insert	F	AGCGCCACTGCCCTGTGTGCCGAGTCAGTTACCAACCTG
		R	ACCTCCTCCATGAGGAATGTGTGGTGACCACGATGCTTC
	Vector	F	ACATTCCTCATGGAGGAGGTTGC
		R	GCACACAGGGCAGTGGCG
Tb1Lu-(C-L2)	Insert	F	ACCGTGGTCACCACACATTCCTGGTGGAGGAGGTTGCC
		R	TGTAGCATCCCTTTCAGGTCAGGAACTTGGAACATTCTTCTTTGTTTC
	Vector	F	GACCTGAAAGGGATGCTAC
		R	GAATGTGTGGTGACCACG
q-PCR			
MLV-eGFP		F	CTGCTGCCCGACAACCAC
		R	TCACGAACTCCAGCAGGAC
		Probe	6FAM-CCAGTCCGCCCTGAGCAAAGACC-TAMRA

### Virus production and infection

Tester viruses and Moloney MLV-based gene delivery viruses were produced by transfecting VSV-Genv-, Gag-Pol-, and transgene-expressing plasmids into HEK293FT cells, as described previously (11). For GaLV production, the gag-pol-expressing pczGALVg/p plasmid (kindly provided by Dr. Jonathan P. Stoye) was included with the VSVGenv and pczCFG2-GFP plasmids (64). Retroviral infectivity was examined by titrating tester viruses into target cells, as described previously (11).

### Quantitative PCR

The stages of retroviral RT in infected cells were examined using quantitative PCR to detect late RT products and proviruses, as described previously (11). Primers and probes were designed to detect eGFP encoded by the MLV genome (65, 66) (Table 2).

### Infection of heterokaryocytes

Heterokaryon formation with PEG and infection were performed as described previously (11). Briefly, to produce heterokaryocytes, a mixture of MDTF and bat cells expressing GFP and tdTomato was treated with PEG1500 (Sigma-Aldrich) (11). Successfully fused cells were detected as GFP-tdTomato DP cells using FACS analysis. The cells were then challenged with MLV expressing the E2-Crimson gene and harvested 48 h after challenge. In the FACS analysis, both singlets and doublets were included, so that unfused doublets were analyzed as a PEG-untreated control.

### Immunofluorescence staining

To examine the intracellular distribution of Yangochiropteran TRIM5α, immunofluorescence staining of transduced MDTF cells was performed with V5 tag-specific (clone OZA3, Medical & Biological Laboratories) or FLAG tag-specific (clone L1, BioLegend) antibodies as described previously (11).

### Western blot analysis

Protein expression in the transduced cells was examined using western blotting with a V5 tag- or FLAG tag-specific for bat TRIM5α or a TRIM5α-specific antibody (clone D-6, Santa Cruz Biotechnologies) for primate TRIM5α, as described previously (11). To inhibit proteasome-mediated protein degradation, MG132 (Selleck Biotech) was added to the culture at a final concentration of 1 μg/mL (67), 4 h before cell harvesting. A solvent (dimethyl sulfoxide; Sigma-Aldrich) was added to the control cell culture supernatant.

### Identification of TbraTRIM5-like sequences

To identify TRIM5-like genes in the *T. brasiliensis* genome, BLASTn and tBLASTn searches (68) were run against the *T. brasiliensis* reference genome (GCA_030848825.1; DD_mTadBra1_pri) using TbraTRIM5α (OP272523.1) as a query with options “-word_size 7 -evalue 1e-10” and “-word_size 2 -evalue 1e-1,” respectively. For the BLASTn search, hits comprising at least five exons were retained. Among the tBLASTn hits, loci predicted to contain more than five protein-coding exons and also at least one of them showing over 50% amino acid identity to TbraTRIM5α were retained. These loci were considered candidate TRIM5-like genes.

To identify TRIM5-like transcripts, publicly available mRNA-seq data sets of *T. brasiliensis* (Table S1) were mapped to the *T. brasiliensis* reference genome (GCA_030848825.1; DD_mTadBra1_pri) using HISAT2 v2.2.1 (69) with the option “-k 1” to suppress multimapping and were visualized with Integrative Genomics Viewer (70). Mapped reads of SRR31928647 were extracted and assembled using Trinity v2.14.0 (71). The resultant contigs were used as queries for BLASTn against TbraTRIM5α, and contigs with a BLASTn hit(s) were retained. The longest open reading frames on the retained contigs were extracted and subjected to DeepCoil domain searches (72) and an NCBI Conserved Domain Search (73). Contigs putatively encoding proteins with a canonical TRIM5 domain architecture were considered candidates.

### Phylogenetic analysis of TRIM5 homologs in *T. brasiliensis*

Phylogenetic analyses were performed using TRIM5-like candidates and representative TRIM family genes. The sequences were aligned with MAFFT v7.490 using the E-INS-i algorithm (74). Ambiguously aligned regions were trimmed with ClipKit v1.4.1 (75) using “-m gappy -g 0.01.” The resulting alignments were used for phylogenetic inferences. A maximum-likelihood tree was constructed using RAxML-NG v1.1.0 (76). The best-fit substitution model (HIVb+I+G4) was selected using ModelTest-NG (77). Node support was assessed with 1,000 bootstrap replicates using the transfer bootstrap expectation method (78). Candidate clusters with TRIM5 genes were designated as TRIM5-like genes.

### Immunoprecipitation

To examine the ability of bat TRIM5α to self-ubiquitinate or self-associate, three expression plasmids, V5- and FLAG-tagged TRIM5α and HA-tagged ubiquitin (Addgene #17608; gifted by Dr. Ted Dawson [79]), were co-transfected into HEK293FT cells. After 24 h of incubation, the cleared cell lysates from the transfected cells were mixed with anti-V5-tag mAb-Magnetic Beads (clone OZA3; Medical & Biological Laboratories) according to the manufacturer’s instructions. Antibody-bound proteins were eluted using an elution solution (5% acetic acid in 3% acetonitrile, pH 2.0; Sigma-Aldrich). The eluate was neutralized with 1 volume of 1 M Tris (pH 8.0; Nacalai Tesque) and stored at −80°C until western blot analysis.

### Statistical analysis

Statistical analyses were performed using JASP (version 0.14.1; University of Amsterdam) (80). Statistical significance among groups was assessed using analysis of variance (*P* < 0.05 was considered significant). If these results were not significant, a non-parametric Kruskal–Wallis test was used. When statistical significance was assessed, post hoc comparisons with the control group were performed (Dunnett’s test for Fig. 1A through E, 2A, 2B, 2E, 3B, 3E, 3F, 4D through F, 5B; Fig. S4; S5B and D). Student’s *t*-test was performed for comparisons between groups (Fig. 3D). Results with *P* values of <0.05 (*), <0.01 (**), and <0.001 (***) were considered statistically significant.

## Data Availability

The nucleotide sequences of the newly identified TbraTRIM5α cDNAs have been deposited in NCBI GenBank (accession numbers PX890925, PX890926, and PX890927).

## SUPPLEMENTAL MATERIAL

Supplemental material jvi.01927-25-s0001.docx Descriptive legends for Fig. S1 to S6, supplemental results, and Table S1.

Fig. S1 jvi.01927-25-s0002.tif Amino acid alignment of three TbraTRIM5α variants.

Fig. S2 jvi.01927-25-s0003.tif Protein expression and intracellular distribution of microbat TRIM5α.

Fig. S3 jvi.01927-25-s0004.tif Isolation of TbraTRIM5α-like paralogs from *T. brasiliensis* cell line.

Fig. S4 jvi.01927-25-s0005.tif Titration curves of B-MLV in the presence of bat TRIM5α mutants.

Fig. S5 jvi.01927-25-s0006.tif Effect of the N71S mutation on primate TRIM5α-mediated restriction.

Fig. S6 jvi.01927-25-s0007.tif Ubiquitination of bat TRIM5α.

Fig. S1, continued jvi.01927-25-s0008.tif Amino acid alignment of TbraTRIM5 paralogs.

Fig. S4, continued jvi.01927-25-s0009.tif Titration curves of B-MLV in the presence of bat TRIM5α mutants.

ASM does not own the copyrights to Supplemental Material that may be linked to, or accessed through, an article. The authors have granted ASM a non-exclusive, world-wide license to publish the Supplemental Material files. Please contact the corresponding author directly for reuse.
