# Supplementary material for: Study of retroviral restrictions in a Tadarida bat cell line enlightens specific early blocks and TRIM5 locus multiplication
Source: J Virol. 2026 Mar 16;100(4):e01927-25. doi: 10.1128/jvi.01927-25 (PMC13098238; doi:10.1128/jvi.01927-25)
Supplement: Supplemental material — Descriptive legends for Fig. S1 to S6, supplemental results, and Table S1. [file jvi.01927-25-s0001.docx]

Supplemental Figure captions

**Fig. S1. Amino acid alignment of three TbraTRIM5α variants, related to Fig. 2** Amino acid alignment of TbraTRIM5 variants (A) and three isolated TRIM5-like paralogs (B). Hyphens and asterisks indicate deletions and the conserved residues in three *T. brasiliensis* TRIM5 variants. Each functional domain is indicated by a box.

**Fig. S2. Protein expression and intracellular distribution of Yangochiropteran** **TRIM5**α**, related to Fig. 2** (A) Western blot analysis of Yangochiropteran TRIM5α in stable MDTF cells after G418 selection. Efus, Tbra, and MfulTRIM5αs were tagged with V5, whereas Like1, Like2, and Like3 were tagged with FLAG. TRIM5α proteins were probed with V5-tag-(left panels) or FLAG-tag-(right panels) specific mAbs. TbraTRIM5α was tagged with V5; hence, no specific protein was detected with anti-FLAG mAb (right panels). TbraTRIM5α was detected with anti-V5 mAb (lower panel). Note that the molecular size of TbraTRIM5α_v1 was smaller than that of the other TRIM5α owing to the lack of the B30.2 domain. Asterisks in the left panel indicate specific bands corresponding to TRIM5α proteins. (B) Confocal microscopy showing fluorescence (red in the panels of Efus, Mful, Tbra-v1, and Tbra-V2 TRIM5αs, magenta in the panels of TbraTRIM5-Like1, Like2, and Like3, and green in the panels of Pdas and Rles TRIM5αs; blue, nucleus) of fixed stable bat TRIM5α-expressing MDTF cells grown on coverslips after probing with anti-FLAG mAbs (TbraTRIM5-Like1, Like2, and Like3 panels in the third row) and anti-V5 (other TRIM5αs panels).

**Fig. S3. Isolation of TbraTRIM5α-like paralogs from *T. brasiliensis* cell line, related to Fig. 2** (A) Amino acid similarities between the three TbraTRIM5α-like paralogs and the first isolated TbraTRIM5α. (B) RT-PCR amplification of TbraTRIM5-like homologs from total cellular RNA extracted from untreated or poly(I:C)-treated Tb1.Lu cells (left panel). Representative results from three biological replicates are shown.

**Fig. S4. Titration curves of B-MLV in the presence of bat TRIM5α mutants, related to Fig. 4** (A) Protein expression levels determined using western blotting and B-MLV titration in MDTF cells expressing TbraTRIM5α with single or double point mutations that modify the charges in L1-Bbox2. (B) N-MLV titration in MDTF cell expressing TbraTRIM5α with a double S71N/S87N mutation in L1. (C) Protein expression levels determined using western blotting and MLV titration in MDTF cells expressing RaegTRIM5α with point mutations that change S/N at positions 70 and 86. (D) Protein expression levels determined using western blotting and B-MLV titration in MDTF cells expressing Yangochiropteran TRIM5αs derived from permissive *E. fus* and *M. ful* cell lines with point mutations that change the S/N at positions 71 and 87. For the titration experiments and the statistics, see the legend of Fig. 1. (C) Two RaegTRIM5α variants were included; v1 encodes the entire protein, representing RaegTRIM5α, whereas v12 lacks the B30.2 domain and was included as a control. The two western blot images with anti-V5 mAb were obtained from the same blot, but with a longer exposure for the lower image than for the upper image. In the lower image, asterisks indicate the bands of truncated Raeg_v12 proteins. (D) The residue at position 71 of EfusTRIM5α is asparagine; therefore, it was replaced with serine. (E) Comparison of B- and N-MLV infectivity between MDTF cells stably expressing bat TRIM5α after exchanging S and N residues in L1. The infection experiments were repeated three times independently. For details on statistical analysis, please see Fig. 1 legend.

**Fig. S5. Effect of the N71S mutation on primate TRIM5α-mediated restriction, related to Fig. 5** (A) Amino acid alignment of L1-Bbox2 of mammalian TRIM5α. Each dot represents identical amino acids to huTRIM5α. The 2D structures predicted based on the structures deposited in the Protein Data Bank (PDB; accession numbers 4TKP, 5F7T, 5W9A, and 5IEA) are shown above the alignment. Residues corresponding to S71 and S87 in TbraTRIM5α are highlighted in red boxes. (B and D) Effects of S-to-N mutation at positions 70/71 and 86/87 on hu and rhTRIM5α-mediated restriction. (B) MDTF cells stably expressing huTRIM5α mutants were titrated with B- and N-MLV. (D) U-2 OS cells stably expressing hu (shown in blue) and rhTRIM5α (shown in red) were titrated with HIV-1 and SIVmac. For the titration experiments and statistics, see the legend of Fig. 1. (C) Protein expression of primate TRIM5α and its mutants in human U2-OS cells, as detected using western blotting. The TRIM5α proteins on the membrane were probed with a TRIM5α-specific mAb.

**Fig. S6. Ubiquitination of bat TRIM5α** Effects of S-to-N mutation in bat TRIM5α on self-ubiquitination and self-association. 293FT cells were co-transfected with V5- and FLAG-tagged bat TRIM5α-expressing plasmids and an HA-tagged ubiquitin-expressing plasmid. The cleared cell lysate of the transfectants (lower panels, “Cell lysate”) was immunoprecipitated with anti-V5 tag mAb-conjugated magnetic beads. Co-precipitated proteins on the western blot membrane were probed with V5-, FLAG-, or HA-tag-specific mAbs (upper panels, “IP”). Arrows indicate the expected size of each molecule. The experiments were repeated three times, and representative results are shown. Similar results were obtained from three independent experiments.

Supplemental Results

**Methods**

**Genomic search of Fv1-like elements in the *T. brasiliensis* genome**

To examine whether an Fv1 ortholog is present in the genome of *T. brasiliensis*, the reference genome (GCA_030848825.1; DD_mTadBra1_pri) was analyzed as follows. As *Fv1* is located between *Mfn2* and *Miip* in the Muroidea genome (1, 2), tBLASTn (3) searches were performed on the *T. brasiliensis* genome using the amino acid sequences of MFN2 and MIIP proteins from humans, mice, and bats (*Molossus molossus*) to determine the syntenic region from the putative MIIP to MFN2. BLASTn, tBLASTx, tBLASTn, tFASTx, and tFASTy (4) searches were performed against the extracted sequence of the syntenic region using the Fv1 nucleotide or amino acid sequences as queries (GenBank accession numbers: FJ603554.1, FJ603555.1, FJ603556.1, FJ603557.1, and FJ603558.1).

To detect non-syntenic Fv1-like sequences in the *T. brasiliensis* genome, the whole genome was searched using tBLASTn, tFASTx, and rFASTy with Fv1 amino acid sequences as queries. Fragmented sequence hits in tBLASTn were merged using bedtools (bedtools merge -d 1000) (5). Sequences containing the hits and their flanking 1,000 nucleotides were extracted and further used to search ORFs of more than 1,200 bases using getorf in the EMBOSS package (6).

**Results and Discussion**

**S71N mutation reduces TbraTRIM5α self-ubiquitination**

Considering that the RING domain functions as an E3 ubiquitin ligase (7–10), the two S residues in L1 may affect the proteasomal degradation of TbraTRIM5α. To investigate this, V5- and FLAG-tagged TRIM5α was co-expressed with HA-tagged ubiquitin in 293FT cells. V5-tagged proteins in the cleared cell lysates of transfectants were immunoprecipitated using V5 tag-specific magnetic beads. The FLAG-tagged bat TRIM5α proteins co-precipitated with their V5-tagged counterparts (Fig. S6) indicated efficient self-association of each bat TRIM5α. The detection of ubiquitin revealed that Tbra-S71N was less ubiquitinated than Pdas or TbraTRIM5α (Fig. S6), supporting the requirement of S71 for efficient self-ubiquitination. Nevertheless, as Pdas and TbraTRIM5α were similarly ubiquitinated, neither self-association nor self-ubiquitination activity correlated with MLV restriction. In contrast, each S86N mutant was self-ubiquitinated to an extent similar to that of its wild-type counterpart (Fig. S6, right), suggesting that S86N mutation did not affect TRIM5α’s self-ubiquitination activity. Therefore, the S71N and S86N mutations appear to distinctly influence the anti-MLV activity of TbraTRIM5α. Taken together, the S/N mutations in L1 may affect ubiquitination-mediated self-degradation and reduce MLV infectivity. The precise mechanism of the acquisition of B-MLV-restriction ability by TbraTRIM5α is currently being investigated.

**Absence of Fv1-related sequences in the *T. brasiliensis* genome**

To obtain further scientific evidence to support the absence of Fv1-related sequences in the *T. brasiliensis* genome, we searched Fv1-like nucleotide and amino acid sequences in the syntenic region between two highly conserved *Miip* and *Mfn2* (1, 2) gene loci in the *T. brasiliensis* genome (NCBI GenBank Genome Assembly GCA_030848825.1; DD_mTadBra1_pri) using five Fv1 sequences as queries (GenBank accession FJ603554.1, FJ603555.1, FJ603556.1, FJ603557.1, and FJ603558.1), which did not return Fv1 orthologs. Although a query of the Fv1-like sequences outside the syntenic region in the entire *T. brasiliensis* genome by tFASTx and rFASTy searches using the five Fv1 sequences returned sequences homologous to Fv1 (maximum 42.8% amino acid sequence similarity), they did not contain open reading frames comparable in length to those of Fv1. These observations confirmed the absence of a functional copy of the Fv1-like genes in the *T. brasiliensis* genome.

Supplemental references

1. Young GR, Yap MW, Michaux JR, Steppan SJ, Stoye JP. 2018. Evolutionary journey of the retroviral restriction gene Fv1. Proc Natl Acad Sci U S A 115:10130–10135.

2. Boso G, Buckler-White A, Kozak CA. 2018. Ancient Evolutionary Origin and Positive Selection of the Retroviral Restriction Factor Fv1 in Muroid Rodents. J Virol 92:e00850-18.

3. Camacho C, Coulouris G, Avagyan V, Ma N, Papadopoulos J, Bealer K, Madden TL. 2009. BLAST+: architecture and applications. BMC Bioinformatics 10:421.

4. Pearson WR. 2016. Finding Protein and Nucleotide Similarities with FASTA. Curr Protoc Bioinformatics 53:3.9.1-3.9.25.

5. Quinlan AR, Hall IM. 2010. BEDTools: a flexible suite of utilities for comparing genomic features. Bioinformatics 26:841–842.

6. Rice P, Longden I, Bleasby A. 2000. EMBOSS: the European Molecular Biology Open Software Suite. Trends Genet 16:276–277.

7. Wagner JM, Roganowicz MD, Skorupka K, Alam SL, Christensen D, Doss G, Wan Y, Frank GA, Ganser-Pornillos BK, Sundquist WI, Pornillos O. 2016. Mechanism of B-box 2 domain-mediated higher-order assembly of the retroviral restriction factor TRIM5α. Elife 5:1–26.

8. Keown JR, Yang JX, Douglas J, Goldstone DC. 2016. Characterisation of assembly and ubiquitylation by the RBCC motif of Trim5α. Sci Rep 6:26837.

9. Fletcher AJ, Christensen DE, Nelson C, Tan CP, Schaller T, Lehner PJ, Sundquist WI, Towers GJ. 2015. TRIM 5α requires Ube2W to anchor Lys63‐linked ubiquitin chains and restrict reverse transcription. EMBO J 34:2078–2095.

10. Fletcher AJ, Vaysburd M, Maslen S, Zeng J, Skehel JM, Towers GJ, James LC. 2018. Trivalent RING Assembly on Retroviral Capsids Activates TRIM5 Ubiquitination and Innate Immune Signaling. Cell Host Microbe 24:761-775.e6.

Supplemental Table S1

| Table S1. Accession numbers of RNA-seq datasets of *T. brasiliensis* used to identify TRIM5-like transcripts, related to Fig. S3C. |
| --- |
| SRR13417550 |
| SRR31928647 |
| SRR31928648 |
| SRR31968919 |
| SRR31968920 |
| SRR31968921 |
| SRR31968922 |
| SRR31968923 |
| SRR31968924 |
| SRR636883 |
| SRR636884 |
| SRR636885 |
| SRR9703455 |
