# Supplementary figures and images for: Study of retroviral restrictions in a Tadarida bat cell line enlightens specific early blocks and TRIM5 locus multiplication
Source: J Virol. 2026 Mar 16;100(4):e01927-25. doi: 10.1128/jvi.01927-25 (PMC13098238; doi:10.1128/jvi.01927-25)

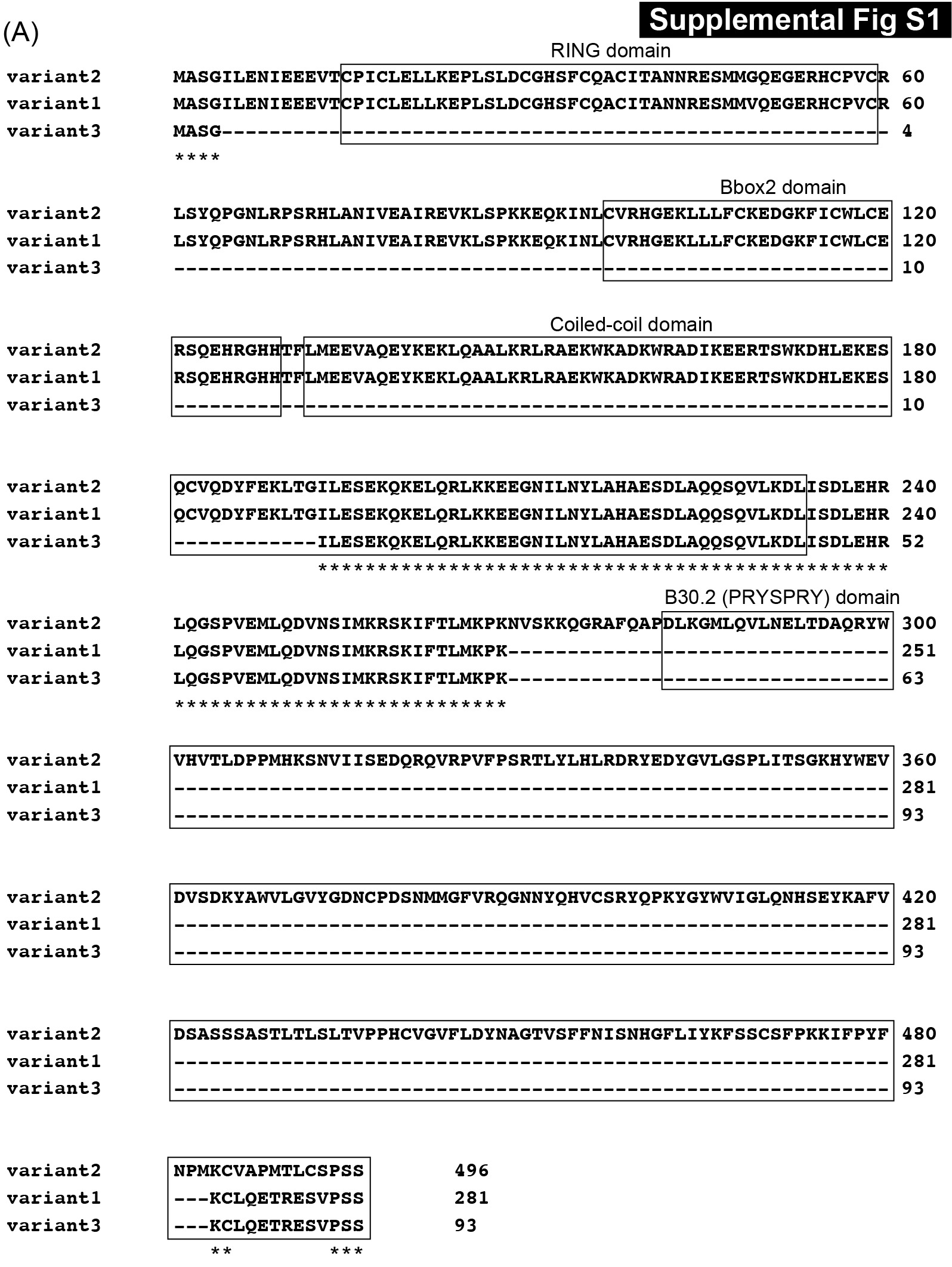

Supplement: Fig. S1 — Amino acid alignment of three TbraTRIM5α variants. [file jvi.01927-25-s0002.tif]

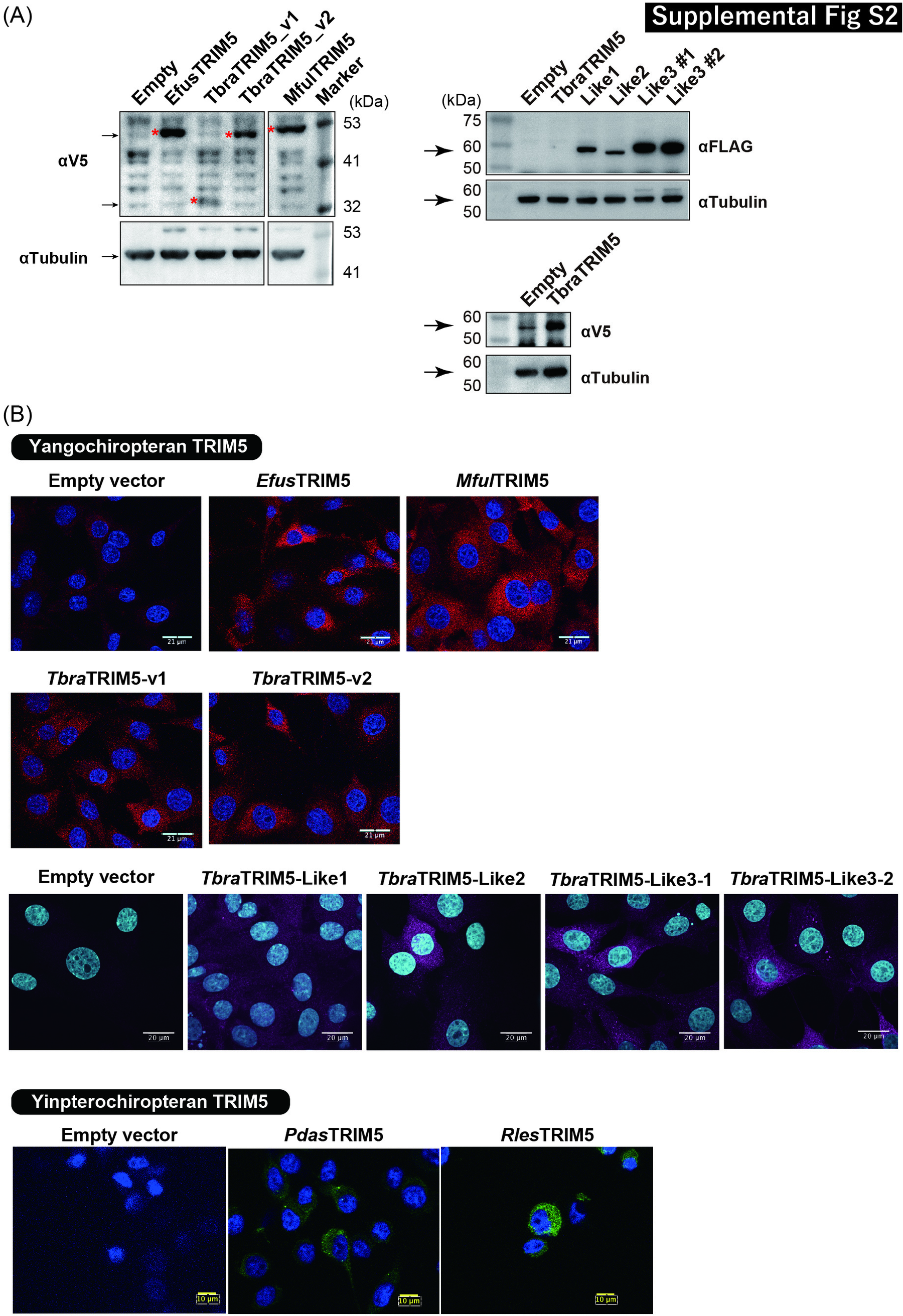

Supplement: Fig. S2 — Protein expression and intracellular distribution of microbat TRIM5α. [file jvi.01927-25-s0003.tif]

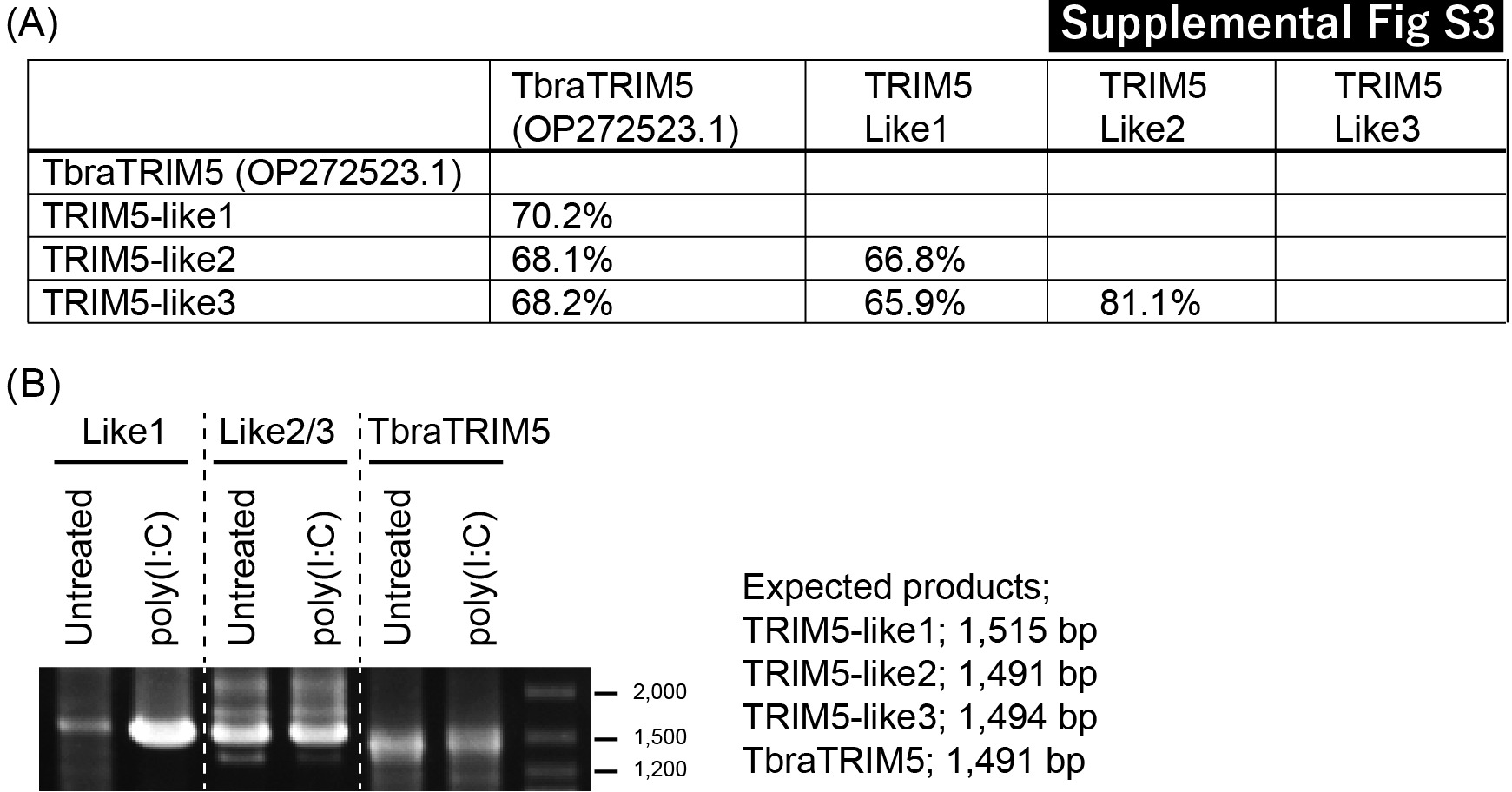

Supplement: Fig. S3 — Isolation of TbraTRIM5α-like paralogs from T. brasiliensis cell line. [file jvi.01927-25-s0004.tif]

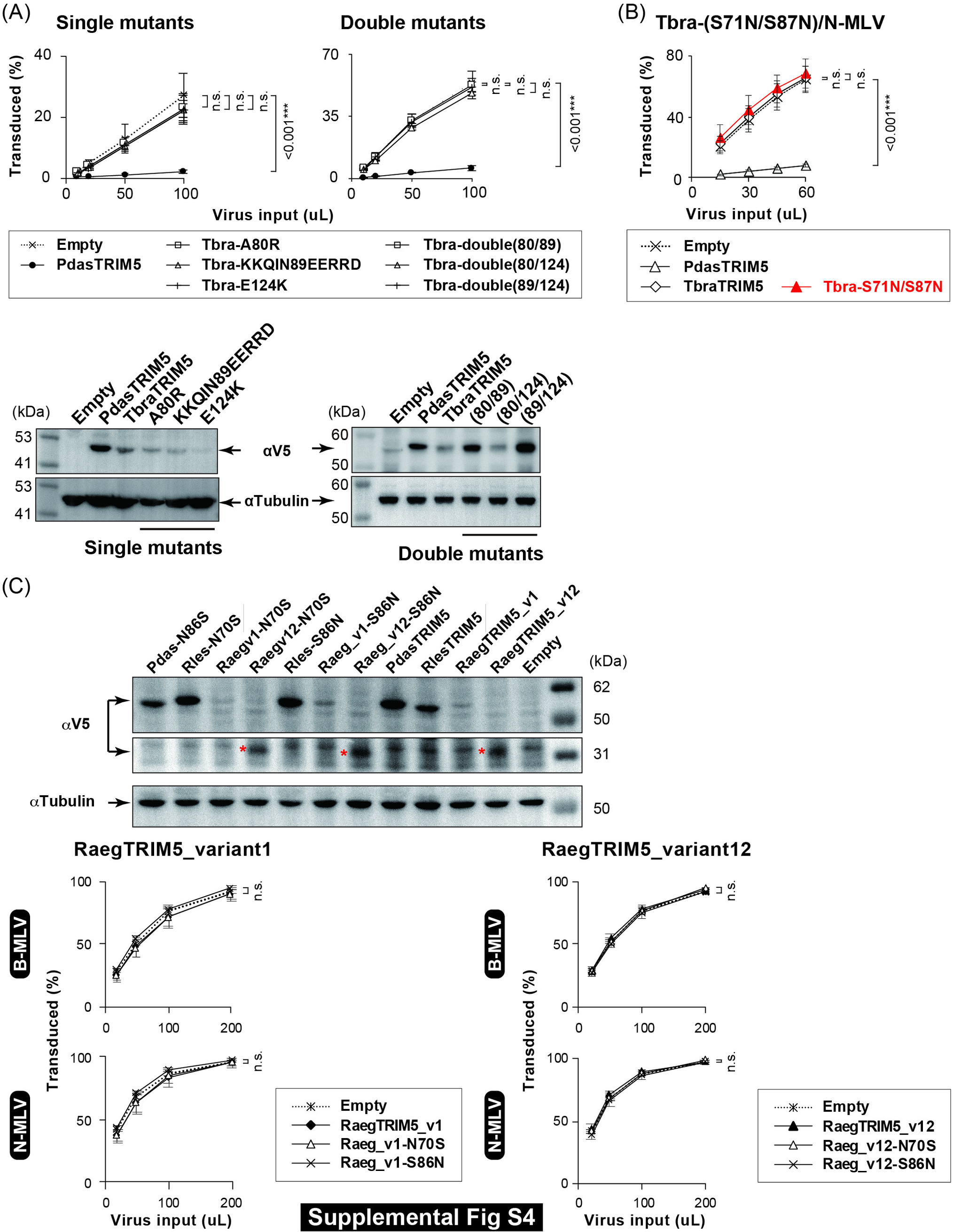

Supplement: Fig. S4 — Titration curves of B-MLV in the presence of bat TRIM5α mutants. [file jvi.01927-25-s0005.tif]

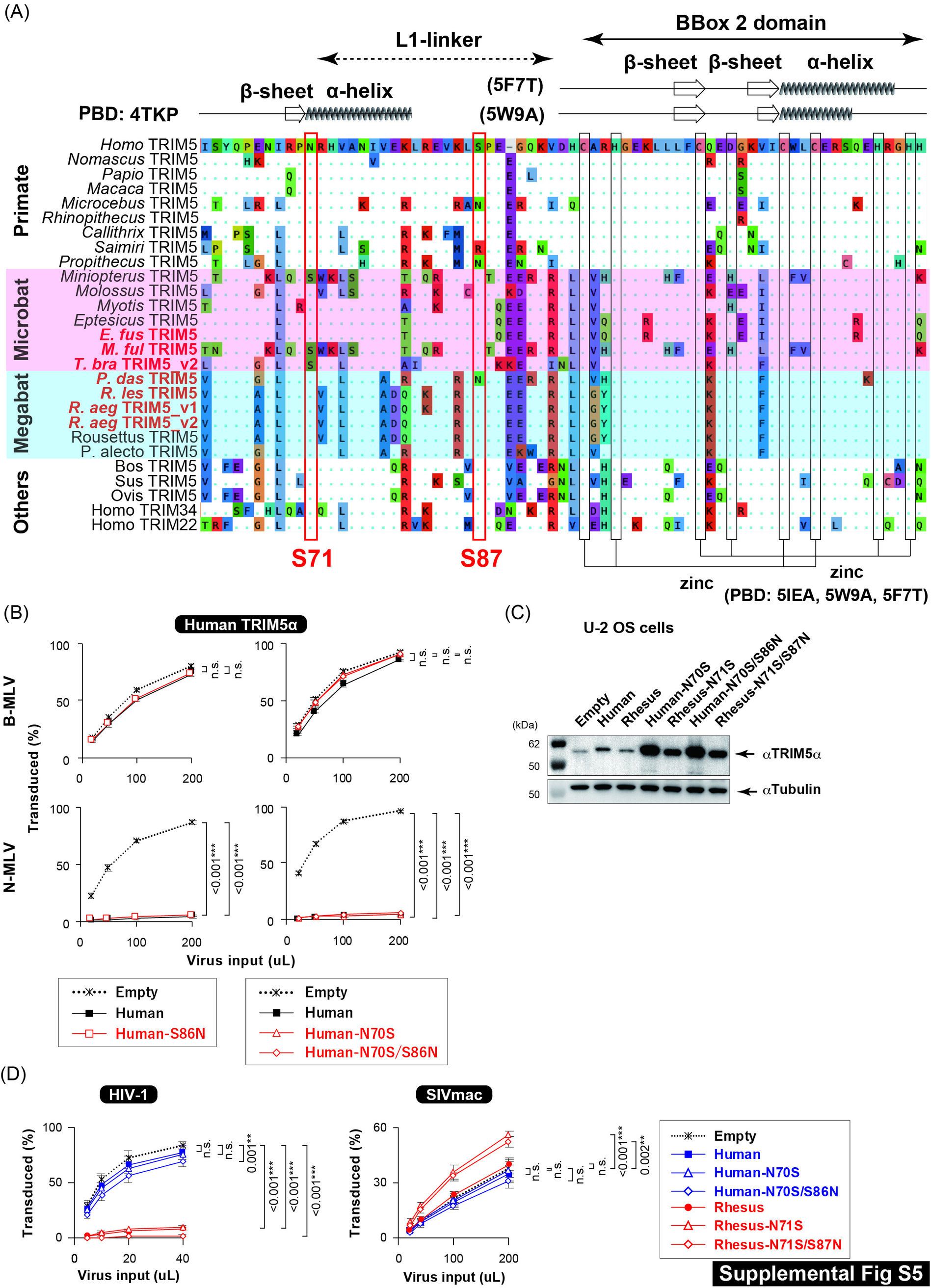

Supplement: Fig. S5 — Effect of the N71S mutation on primate TRIM5α-mediated restriction. [file jvi.01927-25-s0006.tif]

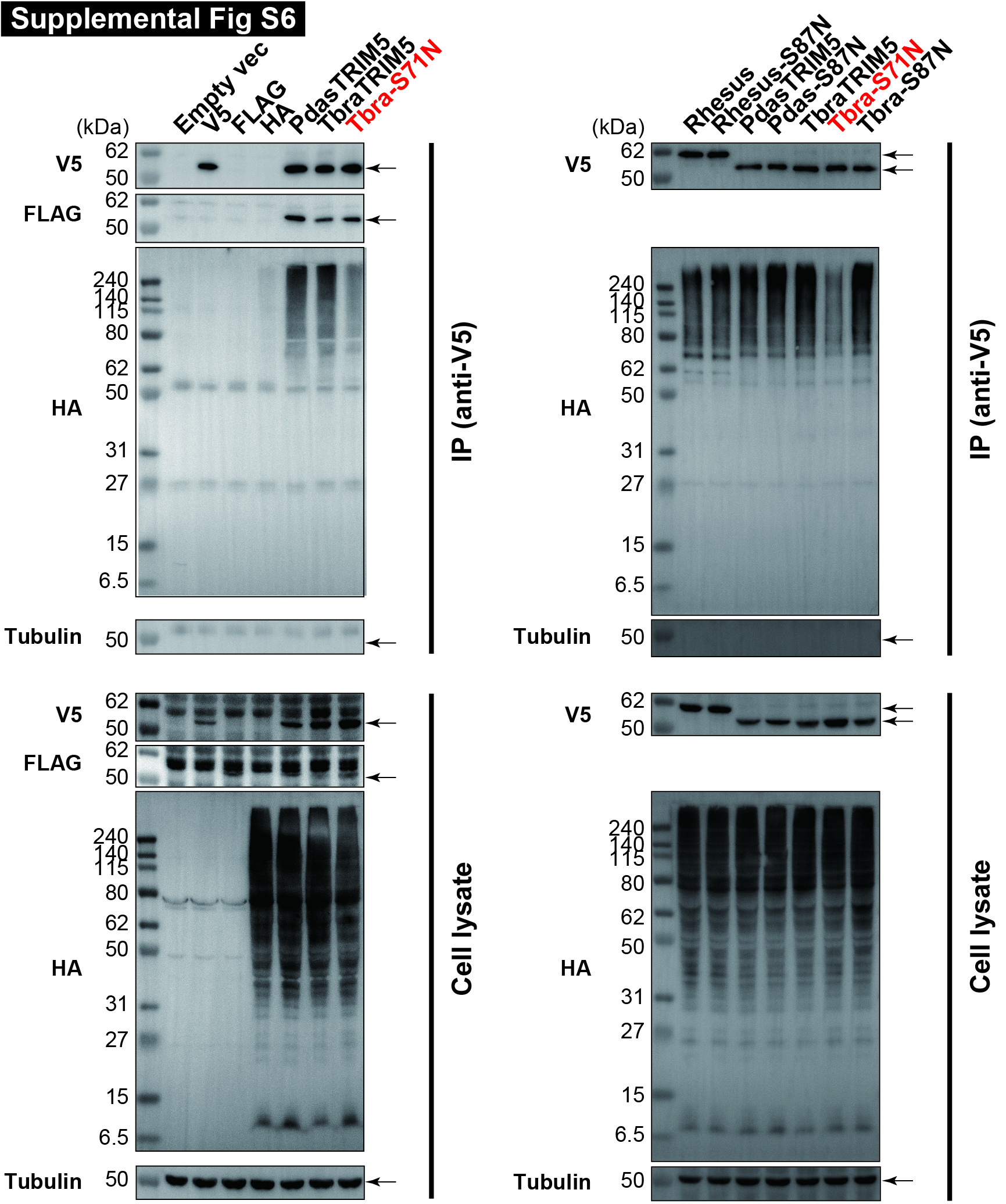

Supplement: Fig. S6 — Ubiquitination of bat TRIM5α. [file jvi.01927-25-s0007.tif]

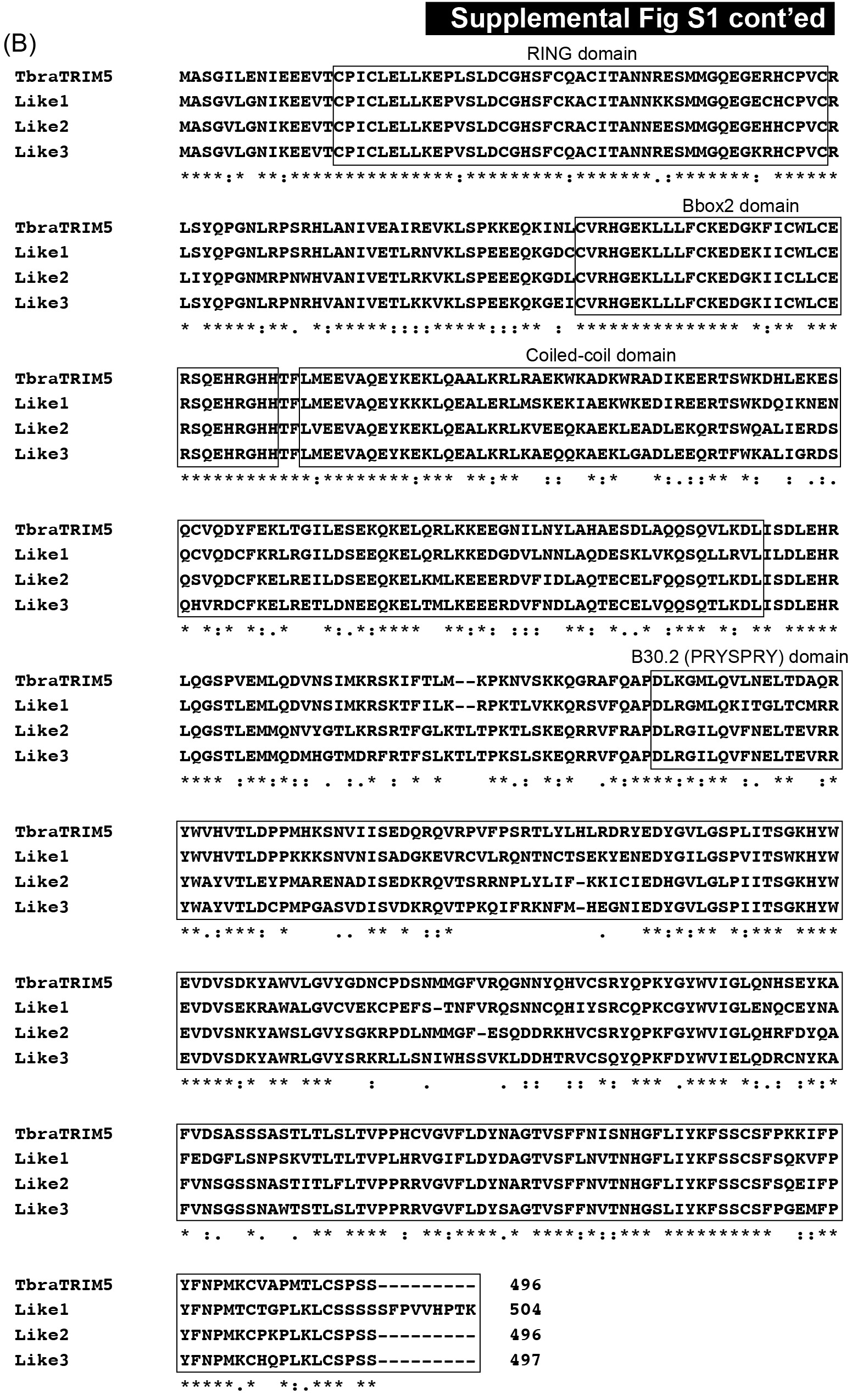

Supplement: Fig. S1, continued — Amino acid alignment of TbraTRIM5 paralogs. [file jvi.01927-25-s0008.tif]

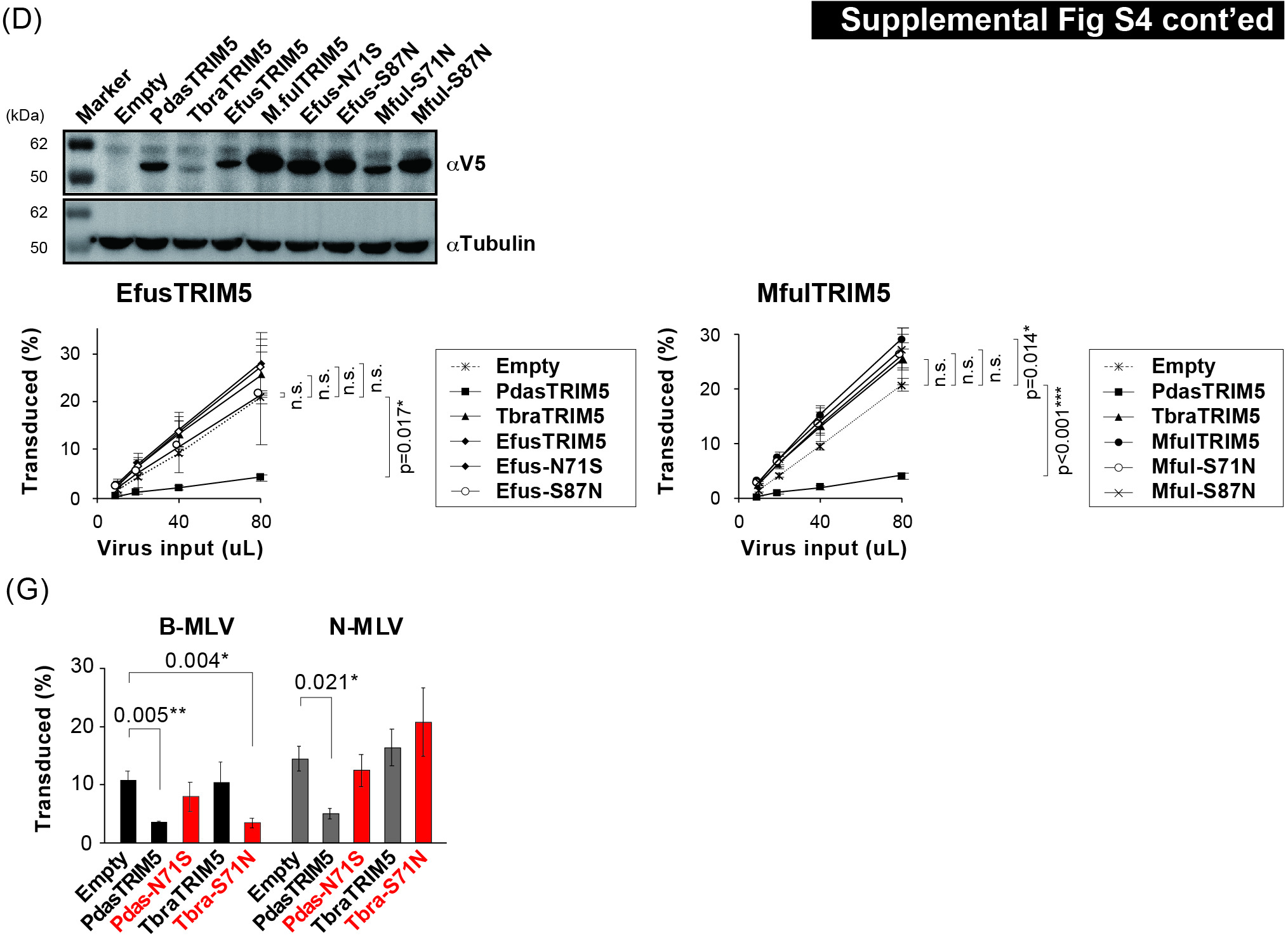

Supplement: Fig. S4, continued — Titration curves of B-MLV in the presence of bat TRIM5α mutants. [file jvi.01927-25-s0009.tif]
